# Identification of marine Important Bird and Biodiversity Areas for penguins around the South Shetland Islands and South Orkney Islands

**DOI:** 10.1002/ece3.4519

**Published:** 2018-10-12

**Authors:** Maria P. Dias, Ana Paula Bertoldi Carneiro, Victoria Warwick‐Evans, Colin Harris, Katharina Lorenz, Ben Lascelles, Harriet L. Clewlow, Michael J. Dunn, Jefferson T. Hinke, Jeong‐Hoon Kim, Nobuo Kokubun, Fabrizio Manco, Norman Ratcliffe, Mercedes Santos, Akinori Takahashi, Wayne Trivelpiece, Philip N. Trathan

**Affiliations:** ^1^ BirdLife International Cambridge UK; ^2^ British Antarctic Survey Natural Environment Research Council Cambridge UK; ^3^ Environmental Research & Assessment (ERA) Cambridge UK; ^4^ Centre for Ecology and Conservation University of Exeter Penryn, Cornwall UK; ^5^ Antarctic Ecosystem Research Division Southwest Fisheries Science Center National Marine Fisheries Service National Oceanic and Atmospheric Administration La Jolla California; ^6^ Division of Polar Life Sciences Korea Polar Research Institute Incheon Korea; ^7^ National Institute of Polar Research Tachikawa Japan; ^8^ Faculty of Science & Technology Anglia Ruskin University Cambridge UK; ^9^ Departamento Biología de Predadores Tope Instituto Antártico Argentino Buenos Aires Argentina

**Keywords:** Antarctica, conservation, marine Important Bird and Biodiversity Areas, penguins, tracking data

## Abstract

**Aim:**

To provide a method of analyzing penguin tracking data to identify priority at‐sea areas for seabird conservation (marine IBAs), based on pre‐existing approaches for flying seabirds but revised according to the specific ecology of *Pygoscelis* penguin species.

**Location:**

Waters around the Antarctic Peninsula, South Shetland, and South Orkney archipelagos (FAO Subareas 48.1 and 48.2).

**Methods:**

We made key improvements to the pre‐existing protocol for identifying marine IBAs that include refining the track interpolation method and revision of parameters for the kernel analysis (smoothing factor and utilization distribution) using sensitivity tests. We applied the revised method to 24 datasets of tracking data on penguins (three species, seven colonies, and three different breeding stages—incubation, brood, and crèche).

**Results:**

We identified five new marine IBAs for seabirds in the study area, estimated to hold ca. 600,000 adult penguins.

**Main conclusions:**

The results demonstrate the efficacy of a new method for the designation of a network of marine IBAs in Antarctic waters for penguins based on tracking data, which can contribute to an evidence‐based, precautionary, management framework for krill fisheries.

## 1 INTRODUCTION

The Important Bird and Biodiversity Area (IBA) program was established by BirdLife International in 1979, with the aim of identifying sites of importance for bird conservation at a global scale (BirdLife International, 2010; Donald, Fishpool, Ajagbe, Bennun, & Bunting, in press; Waliczky, Fishpool, Butchart, Bennun, & Thomas, in press). To date, more than 12,000 IBAs have been documented and delineated worldwide, of which ca. 2,600 have been recognized because of the seabird populations they contain (Donald et al., in press). The delimitation of IBAs was initially focused on terrestrial sites and only began to consider IBAs in marine areas as recently as 2004. The identification of marine IBAs (hereafter mIBAs) was greatly enhanced by the proliferation of scientific studies providing information about the at‐sea distributions of seabirds, especially those based on tracking individual birds (Lascelles et al., 2016).

All mIBAs have been identified using a standardized set of data‐driven criteria and thresholds, ensuring a consistent and comparable approach worldwide (BirdLife International, 2010). To qualify as a mIBA, a site must hold the confirmed regular presence of more than a threshold number of globally threatened species or congregations of one or more species (BirdLife International, 2010 and Supporting information Table S2.2. in Appendix S2). These criteria have been used successfully for many species and have proved effective and versatile in all environments where they have been applied (Donald et al., in press).

One of the main aims of the BirdLife IBA program has been to inform management options and policy responses, through work with national governments, intergovernmental bodies (e.g., European Union), and multilateral environmental agreements (e.g., Convention on Biological Diversity, Ramsar Convention, and Convention of Migratory Species; Waliczky et al., in press). For example, marine IBAs have been designated as Special Protection Areas under the EU Bird's Directive to form part of the Natura 2000 network in a number of countries, including in Spain, Portugal, Italy, Greece, Malta, and Slovenia (Ramírez et al., 2017). Outside of Europe, marine IBAs are informing a range of global and regional policy mechanisms such as the UN World Ocean Assessment, the Convention on Biological Diversity process to describe Ecologically or Biologically Significant marine Areas (EBSAs) in need of protection, the Protocol Concerning Protected Areas and Wild Fauna and Flora in the Eastern African Region to the Nairobi Convention, and the work of the Convention for the Protection of the Marine Environment of the North‐East Atlantic (OSPAR) to define new Marine Protected Areas.

In Antarctica, major advances in the identification of IBAs were made with the identification of 204 terrestrial IBAs, corresponding to the most important breeding colonies for penguins and other seabirds (Harris, Carr, Lorenz, & Jones, 2011; Harris et al., 2015). However, few attempts have been made to identify mIBAs for penguins in Antarctic waters, despite the fact that some major progress has been made in developing statistical tools to define important areas for marine conservation based on tracking data and habitat models (Dias et al., 2017; Lascelles et al., 2016; Soanes et al., 2016) and also in expanding the global databases, such as the Seabird Tracking Database, to include data for penguins (http://seabirdtracking.org/mapper/index.php). One obstacle preventing a more extensive use of these tools for penguins was the fact that most of them have been developed for flying seabirds (e.g., Lascelles et al., 2016), and no attempt had previously been made to adapt the protocols for nonflying seabirds.

This study represents the first attempt to use tracking data to identify marine IBAs for penguins in order to define priority areas for marine conservation in Antarctica. We propose several changes to the existing protocol (published originally in Lascelles et al., 2016) to better reflect the behavior of nonflying seabirds and the quality of tracking data typically available from penguins. These refinements are important because penguins have been identified as of particular conservation concern (Croxall et al., 2012), being one of the most threatened taxa of seabirds with several species showing decreasing trends (BirdLife International, 2018). Moreover, during the breeding season, they have only limited travel capacity in comparison with flying seabirds, and as such, tracking data from penguins may not be well characterized with the previous protocols. We summarize our specific objectives as follows:

1. To develop a method of analyzing penguin tracking data to identify marine IBAs, based on pre‐existing approaches (Lascelles et al., 2016) but adapted according to the specific ecology of *Pygoscelis* penguin species;
2. To test and apply this method to identify an initial portfolio of marine IBAs around the Antarctic Peninsula, South Shetland, and South Orkney archipelagos (The Food and Agriculture Organization of the United Nations [FAO] Subareas 48.1 and 48.2).

## 2 METHODS

### 2.1 Study area, colony information, and tracking data

**Figure 1:**
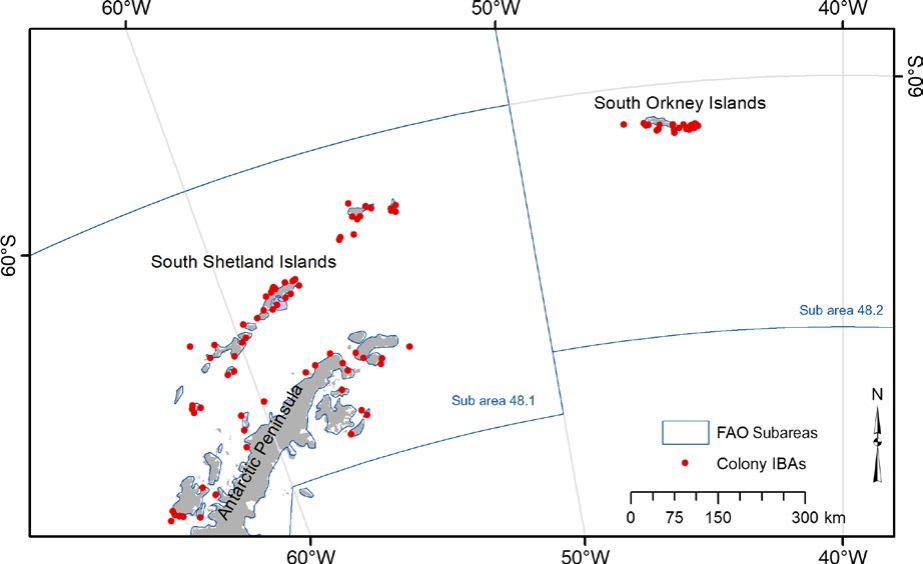
Overview of area of interest showing the Antarctic Peninsula and South Shetland Islands (Subarea 48.1) and South Orkney Islands (Subarea 48.2)

This study focused on the FAO Subarea 48.1, which includes the Antarctic Peninsula and the South Shetland Islands, and on the FAO Subarea 48.2, which includes the South Orkney Islands (Figure 1). A total of 24 “datasets” of tracking data on breeding chinstrap penguin *Pygoscelis antarcticus*, Adélie penguin *Pygoscelis adeliae*, and gentoo penguin *Pygoscelis papua* were available for analysis, provided by 11 contributors (Table 1 and Supporting information Appendix S1). Each dataset corresponds to a unique combination of data collected for a single species in a specific colony, during a unique breeding stage (incubation, brood‐guard, or crèche) and using a certain type of device (Global Positioning System—GPS or platform transmitter terminal—PTT‐Argos). In some cases (mentioned where appropriate), the datasets were further split into different years (Table 1). All datasets were from adult breeding individuals. In total, data for more than 500 different individual birds were compiled, formatted, and stored in the Seabird Tracking Database (http://seabirdtracking.org/mapper/index.php) before analysis.

**Table 1:** Summary of the tracking data analyzed; a complete table with more details of the datasets can be found in Supporting information Appendix S1

Species	Site	Colony	Stage	Device	Sample size (*N* birds)	Used to test	Colony size (pairs)	Source (colony size)
Adélie penguin	South Shetland	Admiralty Bay	Brood	PTT	14		7,032	a
Adélie penguin	South Shetland	Admiralty Bay	Crèche	PTT	41		7,032	a
Adélie penguin	South Shetland	Admiralty Bay	Incubation	PTT	22		7,032	a
Adélie penguin	South Orkney	Powell Island	Brood	PTT	10		49,938	b
Adélie penguin	South Orkney	Signy Island (Gourlay)	Brood	GPS	25		18,333	c
Adélie penguin	South Orkney	Signy Island (Gourlay)	Brood	PTT	24		18,333	c
Adélie penguin	South Orkney	Signy Island (N Point)	Brood	PTT	9		18,333	c
Adélie penguin	Antarctic Peninsula	Hope Bay	Brood	PTT	10		123,850	d
Chinstrap penguin	South Shetland	Admiralty Bay	Brood	PTT	32		950	a
Chinstrap penguin	South Shetland	King George Island	Brood	GPS	48		3,158	e
Chinstrap penguin	South Orkney	Laurie	Brood	GPS	21	1	2,439	f
Chinstrap penguin	South Orkney	Laurie	Incubation	GPS	34	1	2,439	f
Chinstrap penguin	South Orkney	Monroe	Brood	GPS	28	1	33,333	f
Chinstrap penguin	South Orkney	Monroe	Incubation	GPS	13	1	33,333	f
Chinstrap penguin	South Orkney	Monroe	Crèche	GPS	12	1	33,333	f
Chinstrap penguin	South Orkney	Powell	Brood	GPS	34	1	55,213	b
Chinstrap penguin	South Orkney	Powell	Incubation	GPS	13	1	55,213	b
Chinstrap penguin	South Orkney	Signy Island (2013)	Incubation	GPS	9	1	19,530	c
Chinstrap penguin	South Orkney	Signy Island (2015)	Brood	GPS	13	1	19,530	c
Chinstrap penguin	South Orkney	Signy Island (2015)	Incubation	GPS	9	1	19,530	c
Gentoo penguin	South Shetland	Admiralty Bay	Brood	PTT	23		4,736	a
Gentoo penguin	South Shetland	Admiralty Bay	Crèche	PTT	37		4,736	a
Gentoo penguin	South Shetland	King George Island	Brood	GPS	42		2,378	e
Gentoo penguin	South Orkney	Signy Island (North Point)	Incubation	GPS	6		1,315	c

^a^US AMLR program (unpublished data) *in* Lorenz, Harris, Lascelles, Dias, and Trathan (2016). ^b^Poncet and Poncet (1985). ^c^Dunn et al. (2016). ^d^Humphries et al. (2017). ^e^ASPA 171 Management plan *in* Lorenz et al. (2016). ^f^BAS unpublished data.

### 2.2 Data analysis

The analyses were based on a standardized methodology to analyze tracking data that was developed to answer site‐based conservation questions in a repeatable manner (Lascelles et al., 2016). The analysis utilizes a number of different stages, is written in the R language (R Core Team 2016), and uses common functions and packages (Lascelles et al., 2016) to (a) determine hotspots of activity for each individual using kernel density analysis (Wood, Naef‐Daenzer, Prince, & Croxall, 2000), (b) identify boundaries of areas of high‐intensity use by different birds, that is, areas used by more than 10%, 12.5%, or 20% of birds from the colony, depending on the representativeness of the sample (Lascelles et al., 2016); these areas are, at this step, marine IBA candidate sites), (c) determine how representative the tracked population is of the population in the studied colony, (d) predict at‐sea abundances, by multiplying the percentage of birds using the IBA candidate sites by the colony size, and (e) test values against IBA criteria to determine whether an area may qualify as an IBA (detailed at Supporting information Table S2.2 in Appendix S2). This protocol has been tested and applied to more than 80 species, primarily flying seabirds (mostly Procellariiformes), resulting in the identification of more than 500 marine IBAs worldwide (Dias et al., 2017; Lascelles et al., 2016; Soanes et al., 2016).

We modified the marine IBA protocol to make it more suitable for penguins in five ways. First, we changed the method for the interpolation between positions from linear (Lascelles et al., 2016) to one based on continuous‐time correlated random walk models—R package “*crawl”* (Johnson, 2017), which allows interpolation of data at fixed intervals while taking the movement parameters of the individual into account. Second, we removed the “TripSplit” step (Lascelles et al., 2016), as identifying individual foraging trips, especially the short ones, can be virtually impossible with PTT‐Argos‐quality data for Pygoscelis penguins because of the infrequency of observed positions, especially for older datasets. Thus, instead of using different trips as independent observations, we now use all at‐sea location data for each individual bird without splitting into trips (Trathan et al., 2018); this increases the quality of the core areas estimated for each individual and minimizes the risk of pseudoreplication due to individual fidelity to specific foraging sites (Wakefield et al., 2015). Third, we evaluated a range of kernel smoothing factors (*h‐value*). The *h‐value* to use in the kernel analysis of the existing mIBA protocol is usually calculated using a first passage time analysis (Fauchald & Tveraa, 2003; *scaleARS* step in Lascelles et al., 2016), to determine the spatial scales which individuals interact with their environment (Suryan et al., 2006), assuming that the birds have an area‐restricted search behavior (ARS—e.g., Weimerskirch, Pinaud, Pawlowski, & Bost, 2007). However, PTT‐Argos‐based location data from penguins are often unsuitable for ARS estimation since trips and, therefore, within‐trip behaviors cannot be readily identified, due to the typically variable and often low‐accuracy (and infrequent) positions. We tested the performance of the ARS method for penguin tracking data (slightly modified to provide the median scale, rather than the average as was in the original scripts), by comparing the results from the ARS method applied to GPS data only (Table 1) with those obtained by setting fixed *h‐values* that varied between 1 and 10 km, with 1‐km steps (Supporting information Figure S2.2); the maximum value was arbitrary and set based on results obtained in other studies of short‐ranged species (e.g., shags, gulls, and other penguins; Augé et al., 2018). Fourth, we relaxed the constraint of a fixed 50% kernel utilization distribution (UD%) for delineating the core use area of an individual bird (e.g., Soanes, Arnould, Dodd, Sumner, & Green, 2013). The kernel UD50% is usually considered the most appropriate UD% representing the core areas of foraging animals (e.g., Soanes et al., 2013), although some analyses suggest that values around 70% can be more appropriate for penguins (BirdLife International 2009). We compared the results of using UD% between 50% (Lascelles et al., 2016) and 80% (Börger et al., 2006), in 5% increments (Supporting information Figure S2.2). Finally, we analyzed the impact of changing the threshold percentage of the population (PT) used to define the boundaries of the candidate IBA (10%, 12.5%, or 20%, following Lascelles et al., 2016; see above).

Final parameter selection was determined in an iterative process where we ran all potential combinations of *h‐value*, UD%, and PT (231 possible combinations) on a subsample of 10 GPS datasets (five during incubation, four during brood, and one during crèche; Table 1—column “*used to test*”). Each dataset was divided into a *test sample* and a *validation sample* (as described in Supporting information Appendix S2). The tests were carried out using the *test sample*, and the *validation sample* was then used to measure the quality of the final result of each set of values (*h‐value*, UD% and PT). The quality was quantified by analyzing the relationship between the percentage of location data in the *validation sample* that were included inside the candidate IBA site (*inclusion*) and the area of the IBA (Supporting information Figure S2.4 in Appendix S2). The optimum set of parameter values was chosen as the one resulting in the point that minimized the size of the IBA while maximizing the *inclusion* (i.e., the point reaching the asymptote of IBA area‐*inclusion* curve and identified as the first parameter combination resulting in <5% variation in *inclusion*; Supporting information Figure S2.4). Finally, we tested the correlation between the optimum values of *h‐value* and UD% across the different datasets and the maximum distance travelled from the colony (average of the individuals in each dataset). For a detailed explanation of the analysis, see Supporting information Appendix S2.

After identifying the optimum values of the parameters (*h‐value*, UD%, and PT), we applied the new method to a further 14 PTT‐Argos and GPS datasets (Table 1 and Supporting information Appendixes S1 and S2) and checked whether the final sites met the criteria to be classified as marine IBAs (Lascelles et al., 2016; Supporting information Table S2.2 in Appendix S2).

## 3 RESULTS

### 3.1 Tests of the parameters in the marine IBA approach

**Figure 2:**
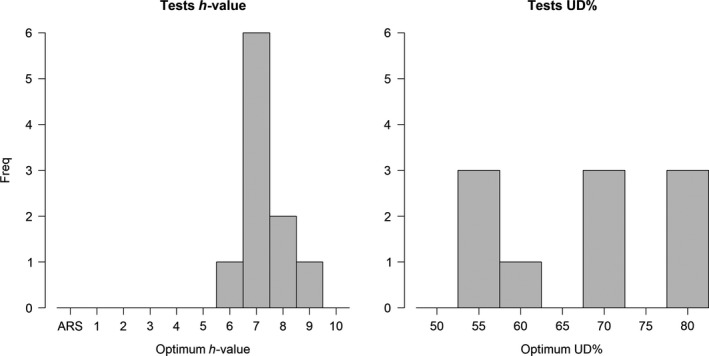
Results of the tests to identify the optimum values for the parameters *h‐value* (left panel) and UD% (right panel) in the kernel analysis. *X*‐axis represents the range of values tested in each parameter (see Methods)

The results of the analyses to identify the optimum *h‐value* and UD% in the kernel analysis (after setting the percentage of birds at 20%, a precautionary value to decrease the risk of overrepresenting the distribution of a single bird in the final results—see Supporting information Appendix S2) revealed a very high consistency of values among the tested samples (Table 2). For the *h‐value*, 7 ± 1 km was almost always the optimum value to choose (Figure 2), irrespective of the breeding stage or colony (Table 2). The *h‐value* resulting from the ARS test was never the optimum value. For the UD%, the optimum values were considerably more variable (Figure 2) and consistently higher during incubation (70%–80%) than during brood (55%; Table 2). The *inclusion* values (percentage of the *validation* sample included in the candidate marine IBAs) were, on average, higher during brood than incubation (82.37% vs 68.48%, respectively; *t*‐test: *t* = 2.96, *df* = 6.71, *p*‐value = 0.0222; Table 2).

**Table 2:** Results of the tests to identify the best values for different parameters (*h‐value* and UD%) in the kernel analyses (based on GPS data) performed to identify candidate marine IBAs for chinstrap penguins breeding at the South Orkney Islands

Colony	Stage	Sample size (birds)	Mean max distance (km)	*h‐value* (ARS)	Best *h‐value* (km)	Best UD%	IBA area (km^2^)	Inclusion value^a^ (%)
Laurie	Incubation	34	34.06	4.29	7	70	759	72.26
Monroe	Incubation	13	126.72	17.43	8	80	5,343	60.95
Powell	Incubation	13	121.54	17.55	9	70	3,669	62.67
Signy2013	Incubation	9	132.89	7.34	7	80	9,340	78.54
Signy2015	Incubation	9	144.96	11.20	7	80	8,932	67.98
Laurie	Brood	21	22.05	3.54	7	55	641	88.86
Monroe	Brood	28	19.58	1.56	7	55	1,056	83.98
Powell	Brood	34	32.73	2.603	6	55	694	83.92
Signy2015	Brood	13	72.00	11.58	7	70	2,394	72.71
Monroe	Crèche	12	54.67	8.22	8	60	1,632	76.76

The *inclusion* value reflects the percentage of positions from a *validation* sample included in the final site.

**Figure 3:**
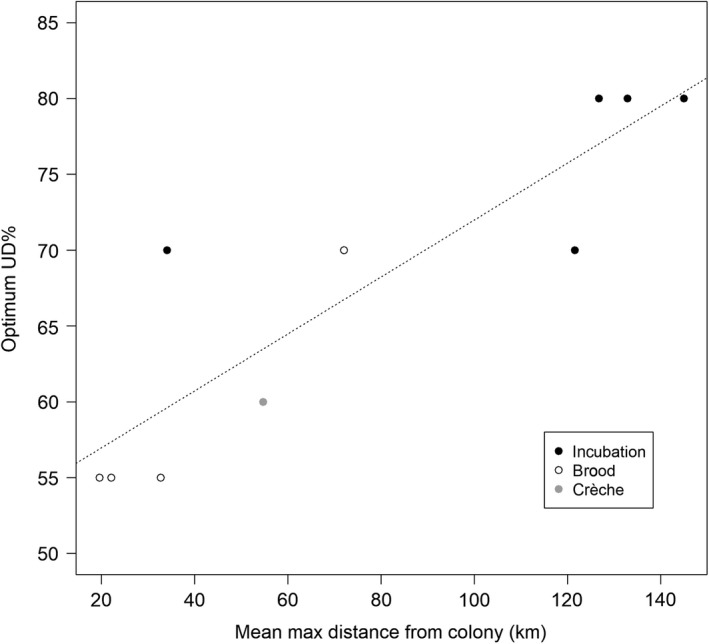
Relationship between the optimum kernel UD% and the maximum distance travelled from the colony. The distance had a significant, positive effect on the optimum UD% (Pearson's correlation *r* = 0.89, *p*‐value = 0.0005, *n* = 10)

**Figure 4:**
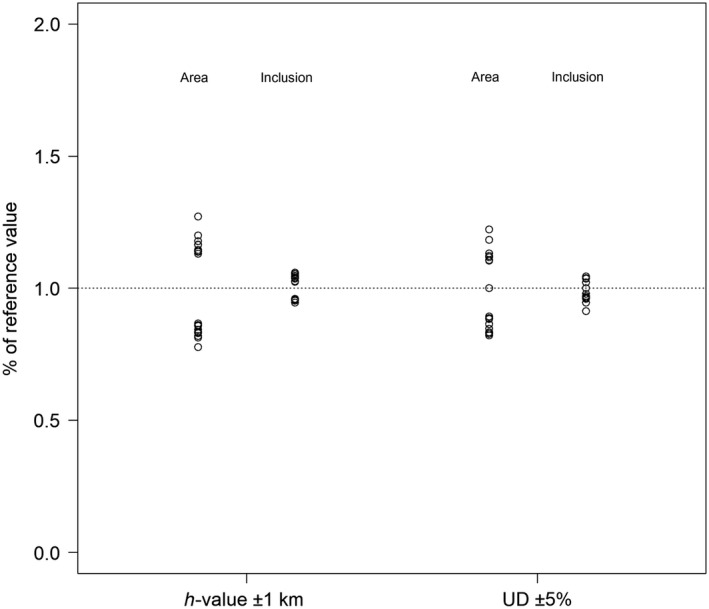
Effect of a change of *h‐value* (on ±1 km) and UD% (±5%) on the final results, measured as the percentage of difference in area and *inclusion* in relation to the reference values (best *h‐value* and best UD%; Table 2)

We found a strong, positive correlation (Pearson's correlation *r* = 0.89, *p*‐value = 0.0005, *n* = 10) between the maximum distance travelled from the colony and the optimum UD% (Figure 3). The distance travelled had no effect on the best *h‐value* (Pearson's correlation *r* = 0.44, *p*‐value > 0.05, *n* = 10). We also found that a small variation in the choice of the *h‐value* (±1 km) or of the UD% (±5%) had little impact on the final results (Figure 4).

### 3.2 Identification of marine IBAs for penguins

Based on the results presented in the previous subsection, we made the corresponding modifications to the current protocol (summarized in Table 3) and applied it to a larger group of datasets of tracking data for penguins (Table 1).

**Table 3:** Summary of the results of the tests to identify the optimum values to use in marine IBA analyses with penguin tracking data

	Incubation	Brood
*h‐value*	7 km	7 km
UD %	75% or as a function^a^ of mean maximum distance	55% or as a function^a^ of mean maximum distance
PT	20%	20%

Function: UD%=mean(maxdist(km))*0.18773 + 53.21025 (Figure 3).

**Figure 5:**
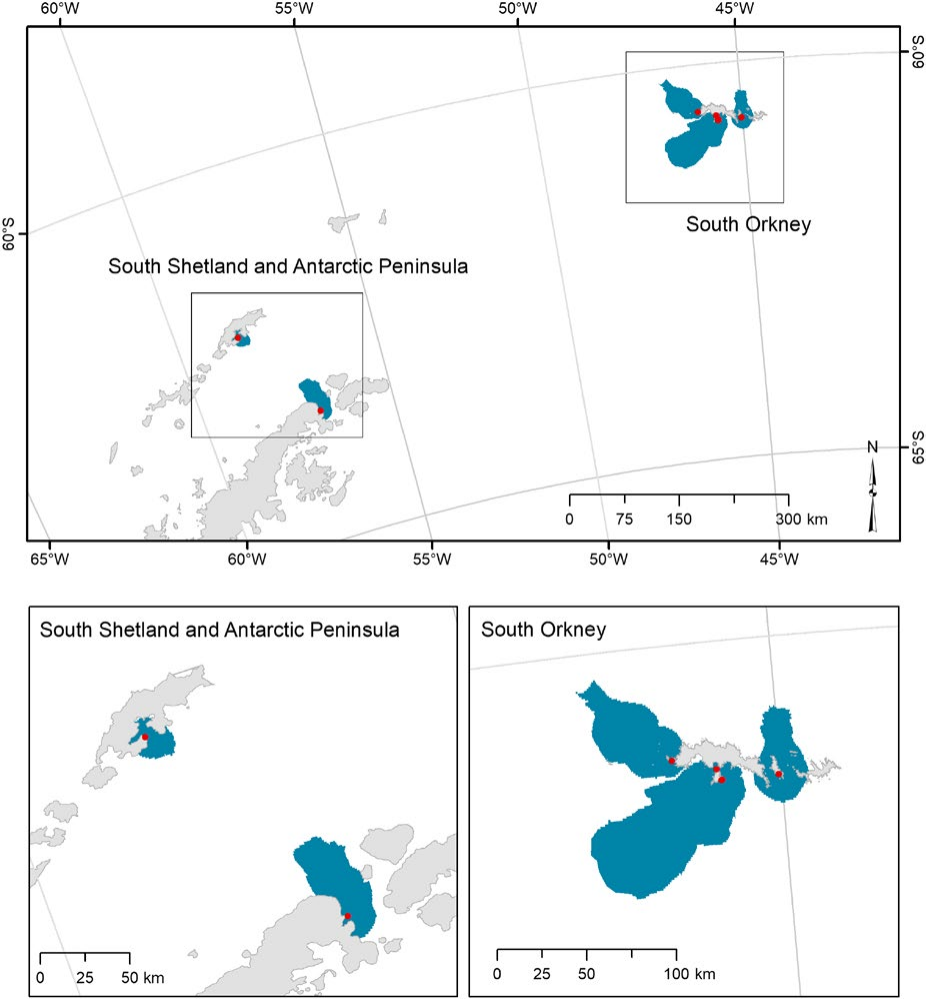
Final marine IBAs confirmed in Subareas 48.1 and 48.2 (in blue), after merging the candidate sites identified for each dataset of tracking data (detailed maps in Supporting information Appendix S3; see also Table 4). Respective colonies represented as red dots

The maps of all candidate marine IBAs identified with the modified protocol can be found in Supporting information Appendix S3. Thirteen of these sites (54%) meet the IBA criteria A4 alone (i.e., due to the presence of a single species), and all except 4 qualified when combined with other IBA candidates identified with data from the same colony (Supporting information Appendix S2). The combination of the layers of the several IBA candidates resulted in the final delineation of five marine IBAs (Figure 5 and Table 4).

**Table 4:** List of marine IBAs identified around colonies located on the Antarctic Peninsula, South Shetland Islands, and South Orkney Islands (FAO Subareas 48.1 and 48.2; see also Figure 5)

Site	IBA criteria	Species	Breed stages	Min population (pairs)^a^	Max population (pairs)^b^
Admiralty Bay	A4iii	Adélie penguin	Incubation, brood‐guard, crèche	1,406	7,032
		Chinstrap penguin	Brood‐guard	190	950
		Gentoo penguin	Brood‐guard, crèche	947	4,736
Monroe	A4ii, A4iii	Chinstrap penguin	Incubation, brood‐guard, crèche	6,667	33,333
Powell	A4ii, A4iii	Adélie penguin	Brood	9,988	49,938
		Chinstrap penguin	Incubation, brood‐guard	11,043	55,213
		Adélie penguin	Brood‐guard	3,667	17,600
Signy	A4iii	Chinstrap penguin	Incubation, brood‐guard	3,906	15,190
		Gentoo penguin	Incubation	263	1,315
Hope Bay	A4iii	Adélie penguin	Brood	24,770	123,850

*Notes*

IBA criteria used: A4ii congregations—site known or thought to hold, on a regular basis, >1% of the global population of a congregatory seabird species (i.e., more than 37,900 pairs of Adélie penguins, 27,000 pairs of chinstrap penguins, or 3,900 pairs of gentoo penguins; values based on Lorenz et al., 2016); A4iii congregations—site known or thought to hold, on a regular basis, >10,000 pairs of seabirds of one or more species.

^a^Based on the minimum percentage of birds using each site, set as 20% (see Methods), multiplied by the colony size (Table 1). ^b^Based on the maximum percentage of birds using each site (depending on the results of the IBA analysis—see Methods and Supporting information Appendix S2), multiplied by the colony size (Table 1).

The marine IBA identified in Antarctic Peninsula (Hope Bay) covers the waters adjacent to the second most important colony of Adélie penguins in the region (after Danger Islands), holding 22% of the total numbers of this species breeding in the region (Antarctic Peninsula east of 60°W; Figure 1; based on data published in the terrestrial IBA inventory; Harris et al., 2015). In the South Orkneys, the marine IBAs identified are located around some of the most important colonies for Adélie penguins (Signy and Powell Islands, holding 27% of the birds breeding in the archipelago) and chinstrap penguins (Monroe, Powell, and Signy Islands, holding 43% of the population breeding there). In the South Shetland, the marine IBA identified along the western shore of Admiralty Bay (King George Island) surrounds one of the major colonies of Adélie penguins (39% of the population) and gentoo penguins (27%) breeding in this archipelago. All together, we estimate that these IBAs cover the most important at‐sea areas of ca. 100,000 pairs of chinstrap penguins, 200,000 pairs of Adélie penguins, and 6,000 pairs of gentoo penguins (Table 4).

## 4 DISCUSSION

This study presents an improvement in the development of a methodological framework to identify priority at‐sea areas of conservation for penguins in Antarctic waters. We tested several changes in previous approaches to apply global criteria to identify foraging hotspots for seabirds, based on tracking data (Dias et al., 2017; Lascelles et al., 2016), and for the first time, we evaluated the quality of the results based on *validation* samples. The major changes in our protocol were related to the interpolation method, now based on a correlated random walk model (Johnson, 2017), which is considered to be more realistic and a better approximation for penguins than assuming linear travel between fixes (Warwick‐Evans et al., 2018), and with two parameters for the kernel density estimates (*h‐value* and the kernel UD%).

By testing several possible combinations of kernel parameter values, we found a remarkable consistency among datasets on the optimum values, especially for the *h‐values* (Table 2). Moreover, we showed that the result of the ARS analysis (used previously to identify the *h‐value*; Lascelles et al., 2016) is of little value for chinstrap penguins (and potentially for other Pygoscelis penguins, but we should note that our tests focused only on GPS data for chinstrap penguins). Even in datasets with very different characteristics (e.g., maximum distance travelled from the colony ranging between 20 and 145 km), the optimum *h‐value* is always around 7 km (6–9 km; Figure 2), and the results are not sensitive to variations around these values (Figure 4). This finding is in line with what was previously shown by other authors analyzing tracking data for penguins (e.g., Trathan et al., 2008). Regarding the UD%, we found a strong correlation between the optimum values and the maximum distance travelled from the colony (Figure 3), which in turn is also related to the breeding stage (Table 2). Penguins, as well as many other seabirds, tend to travel much further during incubation than during brood (e.g., Kato, Yoshioka, & Sato, 2009), so different values of optimum UD% are also suggested for different stages (70%–80% for incubation and 55% for brood).

In general, the results of the tests (as measured by the percentage of the *validation* samples included in the final IBA sites) were better during brood, which is also a reflection of the shorter foraging trips carried out while rearing a chick than while incubating (e.g., Kato et al., 2009). With a smaller foraging area accessible during brood, the overlap between different individuals is necessarily higher, facilitating the identification of areas consistently used by 20% of the tracked population (as required by the IBA analysis; see Methods).

The application of the modified scripts to a set of 24 datasets collected from seven colonies located around the Antarctic Peninsula, South Shetland Islands, and South Orkney Islands (FAO Subareas 48.1 and 48.2) resulted in the identification of five marine IBAs, after merging of overlapping candidate sites (resulting from data collected at the same colony). This constitutes the first set of marine IBAs identified in the region for penguins and, in total, covers an estimated number of more than 300,000 pairs. We should highlight, however, that the application of this method is only possible around colonies where tracking data have been collected. This is an obvious limitation of the method and is particularly relevant to species and sites that are, for logistic reasons, more difficult to track (e.g., many sites and seabird colonies in Antarctica). The future adoption of habitat models to identify priority sites for conservation around important colonies for which tracking data are not available can help overcome this limitation (e.g., Wakefield et al., 2017; Trathan et al., 2018).

## 5 CONCLUSIONS

In this study, we have shown that previous approaches developed for flying seabirds (Lascelles et al., 2016) can be successfully used to identify important marine IBAs around colonies of more range‐restricted, nonflying species, such as penguins. Given recent advances in animal tracking technology, and consequential exponential increases in the number of tracking studies and data availability, a growing number of approaches to analyze data and identify foraging hotspots and key ecological questions for marine taxa have been proposed (Hays et al., 2016). Few attempts, however, have been made to align methodologies across taxa and regions, by having a standardized protocol based on global criteria. The method proposed here was able to identify key at‐sea areas that are a major priority for marine conservation at a global scale (Donald et al., in press). We have shown that the methodology for identifying marine IBAs based on tracking data (Lascelles et al., 2016) can be easily adapted to meet the specific characteristics of the movement of different taxa, providing a robust framework to identify hotspots for multiple species, a fundamental step in the conservation planning processes (Lascelles, Langham, Ronconi, & Reid, 2012). The application of this methodology more broadly can therefore help identify marine IBAs around several other important penguin colonies in Antarctica when tracking data become available, which, in turn, can represent an improved basis for a precautionary, but evidence‐based, management of fisheries in the region.

## CONFLICT OF INTEREST

None declared.

## AUTHOR CONTRIBUTIONS

MPD, CH, BGL, and PT conceived the ideas; MPD coordinated the delivery, analyzed the data, and led the writing; HC, MD, JTH, J‐HK, NK, FM, NR, MS, AT, WT, and PT contributed with tracking data; all authors contributed to the text.

## DATA ACCESSIBILITY STATEMENT

The data used in the analyses can be consulted in the Seabird Tracking Database (www.seabirdtracking.org).

## References

[ece34519-bib-0001] Augé, A. A. , Dias, M. P. , Lascelles, B. , Baylis, A. M. M. , Black, A. , Boersma, P. D. , … Croxall, J. P. (2018). Framework for mapping key areas for marine megafauna to inform Marine Spatial Planning: The Falkland Islands case study. Marine Policy, 92, 61–72. 10.1016/j.marpol.2018.02.017

[ece34519-bib-0502] BirdLife International (2009). Draft guidelines for using seabird tracking data to inform the identification of marine IBAs. Results from a workshop held in CNRS, Chize, France, July 2009. BirdLife international report. Cambridge, UK

[ece34519-bib-0002] BirdLife International (2010). Marine Important Bird Areas toolkit: standardised techniques for identifying priority sites for the conservation of seabirds at sea. Cambridge UK: BirdLife International. Version 1.2: February 2011.

[ece34519-bib-0003] BirdLife International (2018). IUCN Red List for birds. Retrieved from http://www.birdlife.org on 27/07/2018

[ece34519-bib-0004] Börger, L. , Franconi, N. , De Michele, G. , Gantz, A. , Meschi, F. , Manica, A. , … Coulson, T. (2006). Effects of sampling regime on the mean and variance of home range size estimates. Journal of Animal Ecology, 75, 1393–1405. 10.1111/j.1365-2656.2006.01164.x 17032372

[ece34519-bib-0005] Croxall, J. P. , Butchart, S. H. M. , Lascelles, B. , Stattersfield, A. J. , Sullivan, B. , Symes, A. , & Taylor, P. (2012). Seabird conservation status, threats and priority actions: A global assessment. Bird Conservation International, 22, 1–34. 10.1017/S0959270912000020

[ece34519-bib-0006] Dias, M. P. , Oppel, S. , Bond, A. L. , Carneiro, A. P. B. , Cuthbert, R. J. , González‐Solís, J. , … Ryan, P. G. (2017). Using globally threatened pelagic birds to identify priority sites for marine conservation in the South Atlantic Ocean. Biological Conservation, 211, 76–84. 10.1016/j.biocon.2017.05.009

[ece34519-bib-1006] Donald, P.F. , Fishpool, L.D.C. , Ajagbe, A. , Bennun, L.A. , Bunting, G. , Burfield, I.J. , Butchart, S.H.M. , Capellan, S. , Crosby, M.J. , Dias, M.P. , Díaz, D. , Evans, M.I. , Grimmett, R. , Heath, M. , Jones, V.R. , Lascelles, B.G. , Merriman, J.C. , O'Brien, M. , Ramirez, I. , Waliczky, Z. , & Wege, D.C. (in press) Important Bird and Biodiversity Areas (IBAs): the development and characteristics of a global inventory of key sites for biodiversity. Bird Conservation International

[ece34519-bib-0503] Dunn, M. J. , Jackson, J. A. , Adlard, S. , Lynnes, A. S. , Briggs, D. R. , Fox, D. , & Waluda, C. M. (2016). Population Size and Decadal Trends of Three Penguin Species Nesting at Signy Island. South Orkney Islands. PLOS ONE, 11, e0164025 10.1371/journal.pone.0164025 27783668 PMC5082682

[ece34519-bib-0008] Fauchald, P. , & Tveraa, T. (2003). Using first‐passage time in the analysis of area‐restricted search and habitat selection. Ecology, 84, 282–288.

[ece34519-bib-0009] Harris, C. M. , Carr, R. , Lorenz, K. , & Jones, S. (2011) Important Bird Areas in Antarctica: Antarctic Peninsula, South Shetland Islands, South Orkney Islands. Final Report for BirdLife International and Polar Regions Department, UK Foreign & Commonwealth Office. Environmental Research & Assessment, Cambridge.

[ece34519-bib-0010] Harris, C. M. , Lorenz, K. , Fishpool, L. D. C. , Lascelles, B. , Cooper, J. , Coria, N. R. , … Woehler, E. J. (2015). Important Bird Areas in Antarctica 2015. Cambridge, UK: BirdLife International and Environmental Research & Assessment Ltd..

[ece34519-bib-0011] Hays, G. C. , Ferreira, L. C. , Sequeira, A. M. M. , Meekan, M. G. , Duarte, C. M. , Bailey, H. , … Thums, M. (2016). Key questions in marine megafauna movement ecology. Trends in Ecology and Evolution, 31, 463–475. 26979550 10.1016/j.tree.2016.02.015

[ece34519-bib-0500] Humphries, G. R. W. , Naveen, R. , Schwaller, M. , Che‐Castaldo, C. , McDowall, P. , Schrimpf, M. , & Lynch, H. J. (2017). Mapping Application for Penguin Populations and Projected Dynamics (MAPPPD): data and tools for dynamic management and decision support. Polar Record, 53, 160–166. 10.1017/S0032247417000055

[ece34519-bib-0012] Johnson, D. S. (2017). Crawl: fit continuous‐time correlated random walk models to animal movement data. R package version 2.1.1. Retrieved from https://CRAN.R-project.org/package=crawl.

[ece34519-bib-0013] Kato, A. , Yoshioka, A. , & Sato, K. (2009). Foraging behavior of Adélie penguins during incubation period in Lutzow‐Holm Bay. Polar Biology, 32, 181–186. 10.1007/s00300-008-0518-9

[ece34519-bib-0014] Lascelles, B. G. , Langham, G. M. , Ronconi, R. A. , & Reid, J. B. (2012). From hotspots to site protection: identifying Marine Protected Areas for seabirds around the globe. Biological Conservation, 156, 5–14.

[ece34519-bib-0015] Lascelles, B. G. , Taylor, P. , Miller, M. , Dias, M. P. , Oppel, S. , Torres, L. , … Small, C. (2016). Applying global criteria to tracking data to define important areas for marine conservation. Diversity & Distributions, 22, 422–431.

[ece34519-bib-0016] Lorenz, K. , Harris, C. , Lascelles, B. , Dias, M. P. , & Trathan, P. (2016). A first assessment of marine Important Bird and Biodiversity Areas for penguins in Subarea 48.1 (Antarctic Peninsula, and South Shetland Islands) and Subarea 48.2 (South Orkney Islands). Paper submitted to the Working Group on Ecosystem Monitoring and Management, 2016.

[ece34519-bib-0501] Poncet, S. , & Poncet, J. (1985). A survey of penguin breeding populations at the South Orkney Islands. British Antarctic Survey Bulletin, 68, 71–81.

[ece34519-bib-0017] R Core Team (2016). R: A language and environment for statistical computing. Vienna, Austria: R Foundation for Statistical Computing https://www.R-project.org/.

[ece34519-bib-0018] Ramírez, I. , Tarzia, M. , Dias, M. P. , Burfield, I. , Ramos, J. , Garthe, S. , & Paiva, V. (2017). How well is the EU protecting its seabirds? Progress in implementing the Birds Directive at sea. Marine Policy, 81, 179–184. 10.1016/j.marpol.2017.03.034

[ece34519-bib-0019] Soanes, L. M. , Arnould, J. P. Y. , Dodd, S. G. , Sumner, M. D. , & Green, J. A. (2013). How many seabirds do we need to track to define home‐range area? Journal of Applied Ecology, 50, 671–679.

[ece34519-bib-0020] Soanes, L. M. , Bright, J. A. , Carter, D. , Dias, M. P. , Fleming, T. , Gumbs, K. , … Green, J. A. (2016). Important foraging areas of seabirds from Anguilla, Caribbean: Implications for marine spatial planning. Marine Policy, 70, 85–92.

[ece34519-bib-0021] Suryan, R. M. , Sato, F. , Balogh, G. R. , Hyrenbach, K. D. , Sievert, P. R. , & Ozaki, K. (2006). Foraging destinations and marine habitat use of short‐tailed albatrosses: A multi‐scale approach using first passage time analysis. Deep Sea Research Part II: Topical Studies in Oceanography, 53, 370–386.

[ece34519-bib-0022] Trathan, P. N. , Bishop, C. , Maclean, G. , Brown, P. , Fleming, A. , & Collins, M. A. (2008). Linear tracks and restricted temperature ranges characterise penguin foraging pathways. Marine Ecology Progress Series, 370, 285–294. 10.3354/meps07638

[ece34519-bib-1005] Trathan, P. N. , Warwick‐Evans, V. , Hinke, J. T. , Young, E. F. , Murphy, E. J. , Carneiro, A. P. B. , Dias, M. P. , Kovacs, K. M. , Lowther, A. D. , Godø, O. R. , Kokubun, N. , Kim, J. H. , Takahashi, A. , & Santos, M. (2018). Managing fishery development in sensitive ecosystems: identifying penguin habitat use to direct management in Antarctica. Ecosphere, 9, e02392 10.1002/ecs2.2392

[ece34519-bib-0024] Wakefield, E. D. , Cleasby, I. R. , Bearhop, S. , Bodey, T. W. , Davies, R. D. , Miller, P. I. , … Hamer, K. C. (2015). Long‐term individual foraging site fidelity–why some gannets don't change their spots. Ecology, 96, 3058–3074. 27070024 10.1890/14-1300.1

[ece34519-bib-0025] Wakefield, E. D. , Owen, E. , Baer, J. , Carroll, M. J. , Daunt, F. , Dodd, S. G. , … Bolton, M. (2017). Breeding density, fine‐scale tracking, and large‐scale modeling reveal the regional distribution of four seabird species. Ecological Applications, 27, 2074–2091. 10.1002/eap.1591 28653410

[ece34519-bib-0007] Waliczky, Z , Fishpool, L.D.C. , Butchart, S.H.M. , Thomas, D. , Heath, M.F. , Hazin, C , Donald, P.F. , Kowalska, A , Dias, M.P , & Allinson, T.S.M. (in press). Important Bird and Biodiversity Areas (IBAs): 2. the impact of IBAs on conservation policy, advocacy and action. Bird Conservation International

[ece34519-bib-0027] Warwick‐Evans, V. , Ratcliffe, N. , Clewlow, H. , Ireland, L. , Lowther, A. , Manco, F. , & Trathan, P. N. (2018). Using preferred habitat models for chinstrap penguins (*Pygoscelis Antarctica)* to advise krill fisheries management during the penguin breeding season. Diversity and Distribution. 10.1111/ddi.12817

[ece34519-bib-0028] Weimerskirch, H. , Pinaud, D. , Pawlowski, F. , & Bost, C.‐A. (2007). Does prey capture induce area‐restricted search? A fine‐scale study using GPS in a marine predator, the wandering albatross. The American Naturalist, 170, 734–743. 10.1086/522059 17926295

[ece34519-bib-0029] Wood, A. G. , Naef‐Daenzer, B. , Prince, P. A. , & Croxall, J. P. (2000). Quantifying Habitat Use in Satellite‐Tracked Pelagic Seabirds: Application of Kernel Estimation to Albatross Locations. Journal of Avian Biology, 31, 278–286. 10.1034/j.1600-048X.2000.310302.x

