# Supplementary material for: Identification of marine Important Bird and Biodiversity Areas for penguins around the South Shetland Islands and South Orkney Islands
Source: Ecol Evol. 2018 Oct 12;8(21):10520–9. doi: 10.1002/ece3.4519 (PMC6238121; doi:10.1002/ece3.4519)
Supplement: Supplementary file 1 [file ECE3-8-10520-s001.pdf]

Supporting Information 1: DETAILS OF THE TRACKING DATA USED IN THE ANALYSES

Identification of marine Important Bird and Biodiversity Areas for penguins around the South Shetland Islands and South Orkney Islands,
by MP Dias, APB Carneiro, V Warwick-Evans, C Harris, K Lorenz, B Lascelles, H Clewlow, MJ Dunn, JT Hinke, J-H Kim, N Kokubun, F Manco, N Ratcliffe, M Santos,A Takahashi,W Trivelpiece,P Trathan.

Species	Colony	Colony Latitude	Colony Longitude	Breed stage	Device	N Birds	N locations	Years	Contributors	STD ID*
Adélie Penguin	Admiralty Bay	-62.18	-58.442	brood-guard	PTT	14	942	1997-2013	J. Hinke, W. Trivelpiece	910
Adélie Penguin	Admiralty Bay	-62.18	-58.44	crèche	PTT	41	7800	2002-2013	J. Hinke, W. Trivelpiece	910
Adélie Penguin	Admiralty Bay	-62.18	-58.44	incubation	PTT	22	2554	1996-2010	J. Hinke, W. Trivelpiece	910
Adélie Penguin	Hope Bay	-63.37	-56.97	chick-rearing	PTT	10	6425	2013-2014	P. Trathan, M. Santos	753
Adélie Penguin	Powell Island	-60.73	-45.58	chick-rearing	PTT	10	9012	2012-2014	P. Trathan	754
Adélie Penguin	Signy Island (Gourlay)	-60.73	-45.59	brood-guard	GPS	25	10143	2007-2012	N. Ratcliffe, A. Takahashi, P. Trathan	758, 924
Adélie Penguin	Signy Island (Gourlay)	-60.73	-45.59	chick-rearing	PTT	24	5051	1999-2012	M. Dunn, P. Trathan	764,779
Adélie Penguin	Signy Island (North Point)	-60.67	-45.63	chick-rearing	PTT	9	2696	2004-2005	M. Dunn, P. Trathan	773
Chinstrap Penguin	Admiralty Bay	-62.18	-58.44	brood-guard	PTT	32	4945	2005-2013	J. Hinke, W. Trivelpiece	911
Chinstrap Penguin	King George Island	-62.24	-58.79	brood-guard	GPS	48	10194	2006-2015	N. Kokubun, A. Takahashi, J-H Kim	927, 928, 930
Chinstrap Penguin	Laurie	-60.68	-44.59	brood	GPS	21	29085	2011-2012	P. Trathan	759
Chinstrap Penguin	Laurie	-60.68	-44.59	incubation	GPS	28	48142	2011-2012	P. Trathan	759

Species	Colony	Colony Latitude	Colony Longitude	Breed stage	Device	N Birds	N locations	Years	Contributors	STD ID*
Chinstrap Penguin	Monroe	-60.6	-46.06	brood	GPS	28	12772	2015-2016	P. Trathan	NA
Chinstrap Penguin	Monroe	-60.6	-46.06	incubation	GPS	13	20125	2015-2016	P. Trathan	NA
Chinstrap Penguin	Monroe	-60.73	-45.58	crèche	GPS	12	7056	2015-2016	P. Trathan	NA
Chinstrap Penguin	Powell Island	-60.73	-45.58	brood	GPS	34	51442	2013-2014	P. Trathan, A. Lowther	761
Chinstrap Penguin	Powell Island	-60.73	-45.58	incubation	GPS	13	25369	2013-2014	P. Trathan, A. Lowther	761
Chinstrap Penguin	Signy2013	-60.73	-45.59	incubation	GPS	9	21477	2013-2014	N. Ratcliffe, P. Trathan, F. Manco	NA
Chinstrap Penguin	Signy2015	-60.73	-45.59	brood	GPS	13	32150	2015	N. Ratcliffe, H. Clewlow	NA
Chinstrap Penguin	Signy2015	-60.73	-45.59	incubation	GPS	9	17937	2015	N. Ratcliffe, H. Clewlow	NA
Gentoo Penguin	Admiralty Bay	-62.18	-58.44	brood-guard	PTT	23	3354	2004-2013	J. Hinke, W. Trivelpiece	912
Gentoo Penguin	Admiralty Bay	-62.18	-58.44	crèche	PTT	37	8051	2003-2014	J. Hinke, W. Trivelpiece	912
Gentoo Penguin	King George Island	-62.24	-58.79	brood-guard	GPS	42	7417	2006-2015	N. Kokubun, A. Takahashi, J-H Kim	926, 929, 931
Gentoo Penguin	Signy Island (North Point)	-60.67	-45.63	incubation	GPS	6	10421	2013	N. Ratcliffe	762

* STD: Seabird Tracking Database: http://seabirdtracking.org/mapper/index.php; NA: dataset held in the STD but not available online yet
