# Supplementary material for: Identification of marine Important Bird and Biodiversity Areas for penguins around the South Shetland Islands and South Orkney Islands
Source: Ecol Evol. 2018 Oct 12;8(21):10520–9. doi: 10.1002/ece3.4519 (PMC6238121; doi:10.1002/ece3.4519)
Supplement: Supplementary file 2 [file ECE3-8-10520-s002.pdf]

Supporting Information 2: DETAILS OF THE DATA ANALYSIS FOR A PENGUIN-SPECIFIC MARINE IBA PROTOCOL

Identification of marine Important Bird and Biodiversity Areas for penguins around the South Shetland Islands and South Orkney Islands, by MP Dias, APB Carneiro, V Warwick-Evans, C Harris, K Lorenz, B Lascelles, H Clewlow, MJ Dunn, JT Hinke, J-H Kim, N Kokubun, F Manco, N Ratcliffe, M Santos,A Takahashi,W Trivelpiece,P Trathan.

The procedures described below were applied to each tracking dataset (Table 1) used to test and adapt the marine IBA protocol (Lascelles et al., 2016) to penguins. Some steps were applied to all the datasets (n=24; mentioned where relevant). Kernel analyses and area estimates were based on maps projected in a Lambert Azimuthal Equal Area projection, centred in longitude 40°W and latitude 90°S (CRS arguments: "+proj=laea +lat_0=-90 +lon_0=-40 +x_0=0 +y_0=0 +datum=WGS84 +units=m +no_defs +ellps=WGS84 +towgs84=0,0,0").

1. Data preparation and *validation* sample

Both GPS and PTT-Argos data were considered for the IBA analysis (Table 1). Data were cleaned to remove erroneous locations and tracks with fewer than 5 points (PTT-Argos locations were filtered using the *argosfilter* package in R, Freitas 2012). Initially a speed filter was applied to remove positions with speeds >8 km/h (Warwick-Evans et al., 2018). Locations were then interpolated to obtain regular fixes at 5- and 30-minute intervals for GPS and PTT-Argos data (Lascelles et al., 2016; Warwick-Evans et al., 2018), respectively, based on a correlated random walk model using the *crawl* package in R (Johnson 2017). Data were finally cropped to remove locations on land.

Datasets used to test the parameters (all GPS, see Table 1) were split into *test samples* and *validation samples* on the basis of the number and nature of observed complete foraging trips[1] in each dataset. When multiple trips were available for each individual bird (Fig. S2.1), the track (i.e., entire period of deployment of a single bird) was split approximately in half; with the first period considered the *test* and the second the *validation* (vice-versa for half of the birds; Fig. S2.1). When only one entire

[1] The split of samples into trips was only done for the purpose of the tests of the parameters (to split the sample into *test* and *validation*), but not considered in the final IBA identification (where all the data per each individual bird were considered).

trip was recorded per bird (e.g., most incubation tracks), and sample size allowing (n> 10; Table 1), half of the tracks were considered *test* and the other half *validation*. For the remaining datasets (i.e., when multiple trips were not available, or datasets with small sample sizes), the performance of the different parameters (see below) was tested using the original data. Using the original data to test the quality of the parameters had no significant effect on the results (comparison between the *inclusion* values: t = -2.24, df = 7.96, p-value >0.05).

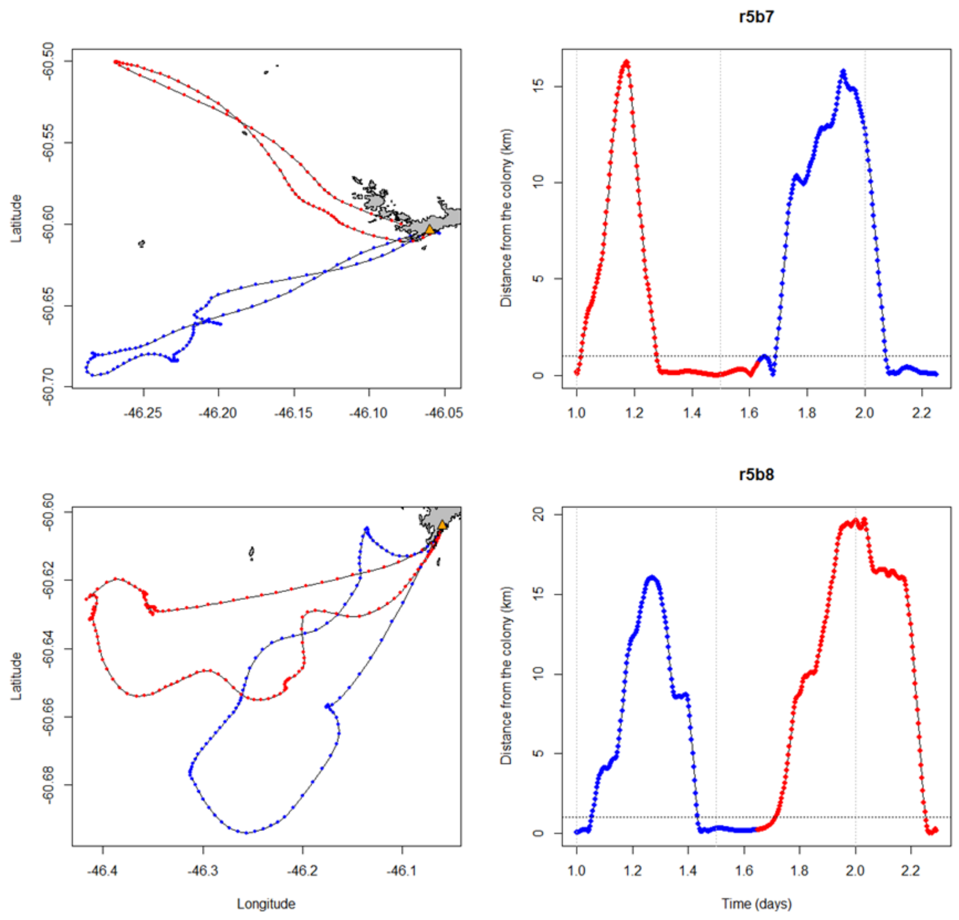

Fig S2.1. Example of the split of the GPS datasets in *test* (blue) and *validation* (red) samples, for tracks with multiple trips. Dashed horizontal line represents a distance to the colony of 1 km.

2. <u>Testing different values for parameters *h-value*, UD percentage and population threshold</u>

Delineation of a marine IBA using the standard protocol (Lascelles et al., 2016) is based on the First Passage Time analysis for setting the *h-value*, and fixed values of UD% and population threshold (PT). Here, we relax those constraints and iteratively evaluate multiple combinations of parameter values to select a final set of parameters that minimizes the size of the marine IBA while maintaining a high

data inclusion percentage[2] (i.e., we've estimated the inflexion point in the curve relating the IBA area and the % of inclusion; see Figure S2.4). Below, we highlight the range of parameter values examined and then describe the parameter selection process. After cleaning and standardising the data, we estimate the core foraging area of each individual bird using a Kernel Density Estimate (Wood et al., 2000), analogous to the *batchUD* determination in marine IBA protocol (Lascelles et al., 2016). The smoothing factor (*h-value*) to use in the kernel analysis is usually calculated in a First Passage Time Analysis (Fauchald and Tveraa 2003; *scaleARS* step in Lascelles et al., 2016), to determine the spatial scales that individuals interact with the environment (Suryan et al., 2006), assuming that the birds have an area-restricted search behaviour (ARS – e.g. Weimerskirch et al., 2007). We tested the performance of the ARS method for penguin tracking data (slightly modified to provide the median scale, rather than the average as was in the original scripts), by comparing the results from the ARS method with those obtained by setting fixed *h-values* that varied between 1 km and 10 km, with 1 km steps (Fig. S2.2); the maximum value was arbitrary and set based on results obtained in other studies of short-ranged species (e.g. Augé et al., 2018).

We also tested the use of different UD%s in the kernel analysis (Börger et al., 2006). The kernel UD50% is usually considered the most appropriate UD% representing the core areas of foraging animals (e.g. Soanes et al., 2013), although some analyses suggest that values around 70% can be more appropriate for penguins (BirdLife International 2009). We compared the results of using UD% between 50% (Lascelles et al., 2016) and 80%, in 5% increments (Fig. S2.2).

After estimating the core area for each individual, the final step in the marine IBA protocol is to identify areas of overlap between different birds (*polyCount* function), and select those areas of overlap that are used by a certain percentage of the tracked individuals (*thresholdRaster* in Lascelles et al., 2016). The areas used by more than this percentage threshold (hereafter PT) are considered candidate IBAs (final confirmation depends on the final step of estimating the number of birds using the area, based on the size of the original colony). This percentage can vary between 10% (i.e. at least 10% of the birds are required to use an area for this to be considered as an IBA candidate), 12.5% or 20%, depending on the result of a bootstrap analysis (Lascelles et al., 2016). We also tested the validity of this approach by comparing the results of using each of these three possible values (Fig. S2.3).

[2] i.e., inclusion percentage = location estimates that fall within the IBA

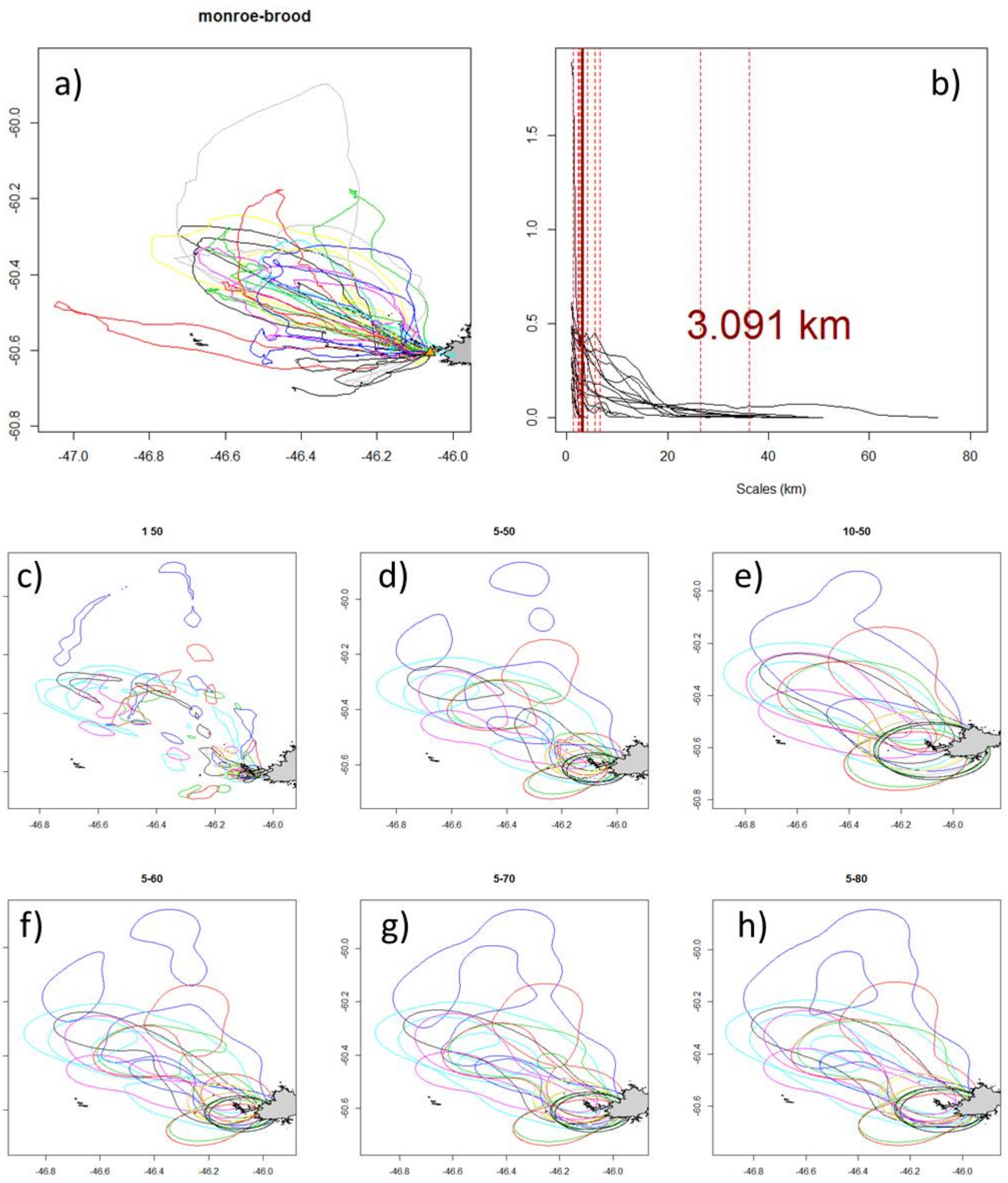

Fig. S2.2: a) Original tracks (*test* sample); b) result of the *ARScale* analysis (Lascelles et al., 2016); c)-e): examples of different results in the kernel analysis to identify the core foraging areas of each individual, by using different *h-values* (1, 5 and 10, respectively; UD% fixed at 50%); f)-h): examples of different results in the kernel analysis to identify the core foraging areas of each individual, by using different UD% (60%, 70% and 80%, respectively, *h-value* fixed at 5 km).

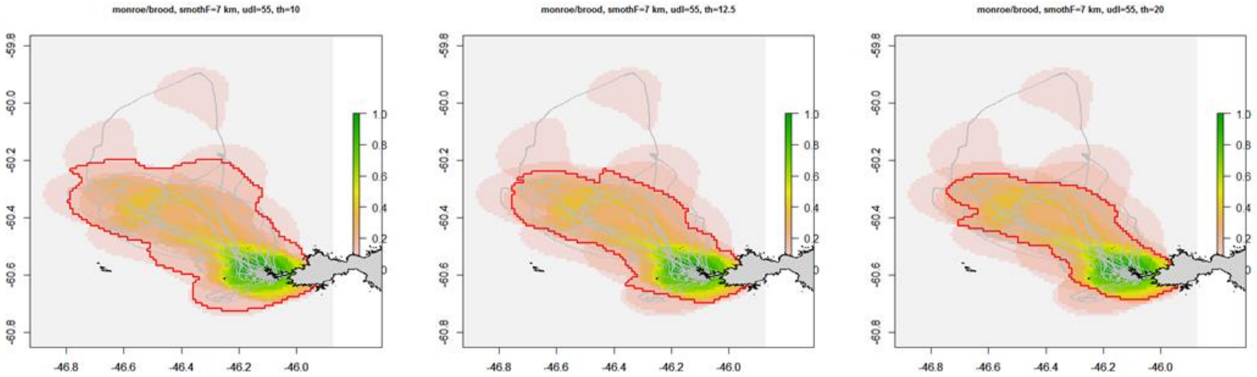

Figure S2.3. Examples of the results of applying different PT values (10%, left panel, 12.5%, middle panel and 20%, right panel), on the dataset collected for Chinstrap penguins from Monroe during brood. Colour gradient represents the proportion of birds using an area. Grey lines represent the tracks.

We tested all possible combinations of these values (11 values of *h-value*, 7 values of UD% and 3 values of PT, resulting in 231 analyses per dataset). We evaluated the quality of the final results by calculating the percentage of points in the *validation* sample included in the IBA candidate (hereafter *Inclusion*), as a function of the final area of the IBA site (Fig. S2.4). We then estimated the coefficient of variation (CV) of the *inclusion* and the area for each parameter (*h-value*, UD% and PT), to identify which had a major impact on the final results. Our results showed that the *h-value* has a major influence on the results (higher values of CV; Figs. S2.5 and S2.6). We therefore carried out an analysis to set the optimum UD% first, and then test the optimum *h-value* given an optimum UD%. This analysis was carried out by analysing the relationship between the IBA area and the *inclusion* (Fig. S2.4 and Fig. S2.6), to find the point that corresponds to the set of values resulting in the smallest possible IBA area, but still representing an increment of more than 5% *inclusion* in comparison with the previous set of points.

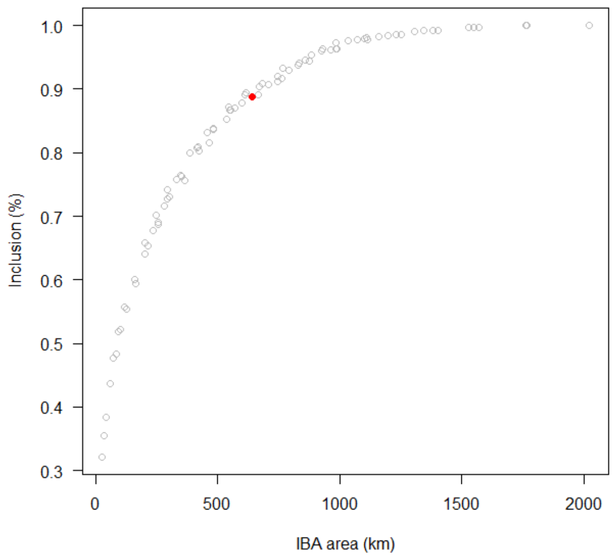

Fig S2.4: Example of the identification of the optimum set of values for the Kernel analyses parameters (dataset: Chinstrap penguin from Laurie Island during brood). The red dot indicates the IBA area that results in an increment of less than 5% in the inclusion of validation points (inflexion point in the curve).

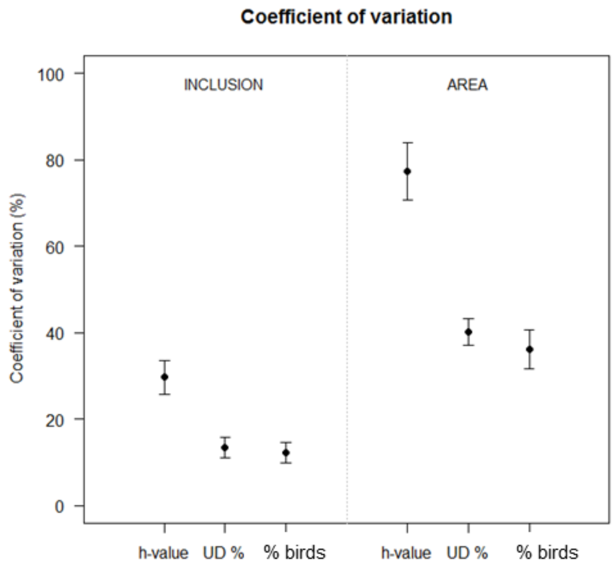

Figure S2.5. Coefficient of variation of the *inclusion* (percentage of the *validation* sample included in the final sites) and of the final area of the sites, in relation to the parameters tested: 1) smoothing factor of the kernel density estimates (*h-value*), 2) UD % of the kernel density estimates and 3) final PT using an area to be considered as a candidate site (%birds). The smoothing factor has a significantly higher impact on the variability of the results (ANOVA test $F_{2,21}$= 10.98, p= 0.0005; Tukey post-hoc test: *h-value* significantly different from both UD% and PT; no differences between UD% and PT).

The tests also revealed that variations in the PT value can have a very large impact on the results for small sample sizes (n< 10; Fig. S2.6; significant correlation between CV of *inclusion* due to PT and sample size; Pearson's correlation R=-0.646; df = 8; p-value = 0.04). This is expected because, for small sample sizes, one individual might represent a large percentage of the sample (10%, if n<10). Therefore we decided to keep these samples but have a precautionary approach of using 20% as a fixed PT, across all datasets, to decrease the risk of over-representing the distribution of a single bird in the final results. All the tests to find the optimum *h-value* and UD% were then made fixing the PT at 20%.

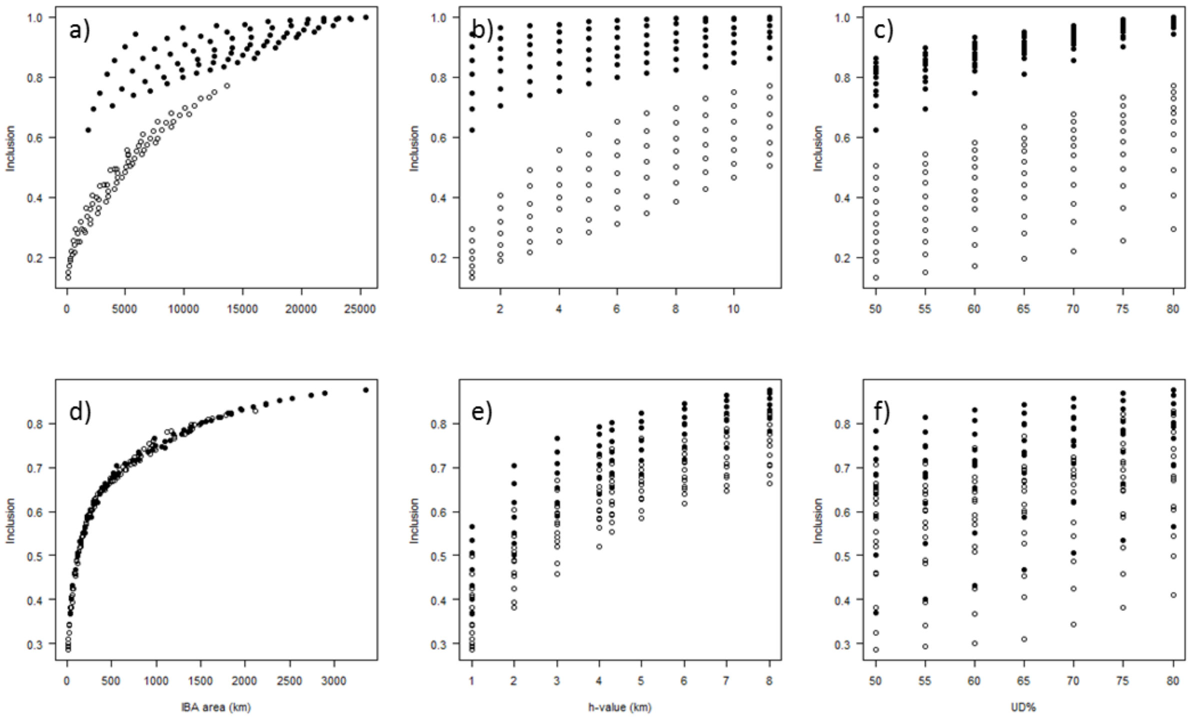

Figure S2.6. Example of the effect of the PT value (closed dots: 10%; open dots: 20%) on the results of the analysis in a dataset with a small sample size (n=9; Chinstrap penguin, Signy2015, incubation, top panels a, b and c), showing that selecting a PT of 10% results in over estimating the *inclusion*, when compared with a dataset with a good sample size (n=34, Laurie, incubation; lower panels d, e and f).

3. Applying the marine IBA approach to other datasets and identifying the final IBA boundaries

After finding the optimum values for the kernel analysis, 14 additional datasets were analysed (Table 1 main text), corresponding in total to 7 colonies and 3 species. The final parameters used in the IBA analyses are summarized in Table S2.1, along with the minimum and maximum percentage of birds using the areas, and the estimates of the final number of birds in each site (maps in Appendix 3). For these analyses, all the data for each individual were considered when identifying the individual core areas (i.e., different trips were not analysed as independent observations as suggested by Lascelles et al., 2016). We also run the representativeness test to access the quality of the datasets (Lascelles et al., 2016); only one had a representatives lower than 70% (1 of the 3 datasets available for Adélie Penguin in Signy Island during brood-guard; Table S2.1), so this dataset was excluded from the list of the candidate sites (Lascelles et al., 2016). The final boundaries of the marine IBAs were then delineated by merging the candidate sites identified from each dataset (given that several datasets corresponded to the same colony, resulting in a considerable overlap between candidate IBAs; Fig. S2.7). In order to estimate the final number of birds of each species using the IBA, we considered the maximum abundance within breeding stages (when more than one was available; see example for Powell Island, Table S2.1 and Fig. S2.7). Finally, we checked the IBA criteria (Table S2.2) for each final site.

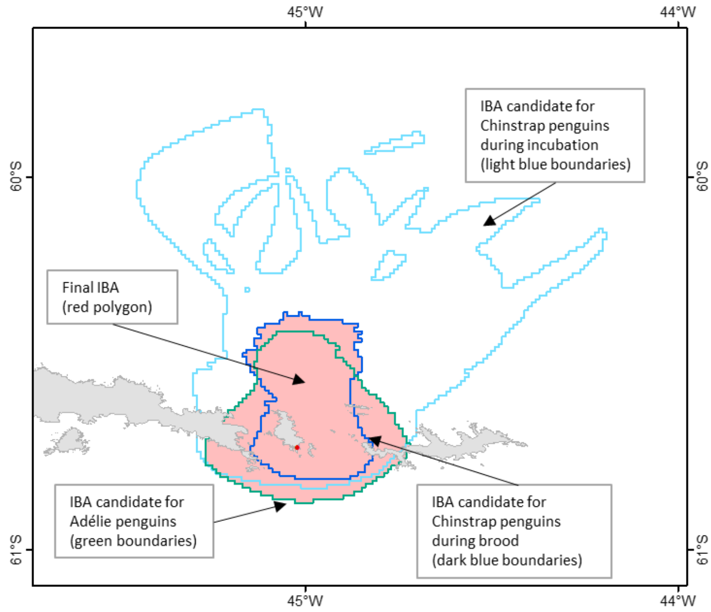

Fig. S2.7: Example of how the candidate sites for several datasets from the same colony were merged (Powell Island, South Orkney Islands). Multiple sites identified for the same species were first merged (in the example, Chinstrap penguins during brood and incubation), giving preference to the sites identified during brood (given the higher quality of these sites and the fact that these are usually included in the sites identified during incubation; Table 2 in main text). Sites for different species were then merged to obtain the final IBA polygon (red area in the map).

Table S2.1: IBA analyses for the 24 datasets (see also Table 1, main text).

Species	Site	Colony	Device	Breeding stage	Sample size	Mean max distance to colony (km)	*h-value*	UD (%)	Candidate IBA area (km²)	Max. % of birds in cand. IBA	Min. pop. estimate (pairs)[1]	Max. pop. estimate (pairs)[2]	Representa-tiveness (%)
Adélie Peng.	South Shetland	Admiralty Bay	PTT	brood-guard	14	26.33	7	55	658	100	1406	7032	98.37
Adélie Peng.	South Shetland	Admiralty Bay	PTT	crèche	41	167.23	7	60	614	90.24	1406	6346	97.03
Adélie Peng.	South Shetland	Admiralty Bay	PTT	incubation	22	200.37	7	75	1466	81.82	1406	5754	91.8
Adélie Peng.	Antarctic Peninsula	Hope Bay	PTT	brood-guard	10	79.84	7	60	1593	100	24770	123850	96.94
Adélie Peng.	South Orkney	Powell Island	PTT	brood-guard	10	433.7	7	60	994	100	9988	49938	90.83
Adélie Peng.	South Orkney	Signy Island (Gourlay)	GPS	brood-guard	25	13.56	7	55	425	96	3667	17600	98.37
Adélie Peng.	South Orkney	Signy Island (Gourlay)	PTT	brood-guard	24	148.58	7	60	1660	79.17	3667	14514	85.89
Adélie Peng.	South Orkney	Signy Island (N Point)	PTT	brood-guard	9	471.27	7	60	3624	77.78	3667	14259	NA[4]
Chinstrap Peng.	South Shetland	Admiralty Bay	PTT	brood-guard	32	20.59	7	55	438	100	190	950	99.98
Chinstrap Peng.	South Shetland	King George Island	GPS	brood-guard	48	18.31	7	55	608	83.33	632	2632	97.79
Chinstrap Peng.	South Orkney	Laurie	GPS	brood	21	22.05	7	55	641	-[3]	-	-	99.23
Chinstrap Peng.	South Orkney	Laurie	GPS	incubation	28	34.06	7	70	759	-	-	-	97.13
Chinstrap Peng.	South Orkney	Monroe	GPS	brood	28	19.58	7	55	1056	100	6667	33333	98.22
Chinstrap Peng.	South Orkney	Monroe	GPS	incubation	13	126.72	8	80	5343	92.31	6667	30770	83.36
Chinstrap Peng.	South Orkney	Monroe	GPS	crèche	12	54.67	8	60	1632	91.67	6667	30556	86.13
Chinstrap Peng.	South Orkney	Powell Island	GPS	brood	34	32.73	6	55	694	100	11043	55213	99.11
Chinstrap Peng.	South Orkney	Powell Island	GPS	incubation	13	121.54	9	70	3669	100	11043	55213	89.5
Chinstrap Peng.	South Orkney	Signy2013	GPS	incubation	9	132.89	7	80	9340	77.78	3906	15190	77.79
Chinstrap Peng.	South Orkney	Signy2015	GPS	brood	13	72	7	70	2394	100	3906	19530	94.31
Chinstrap Peng.	South Orkney	Signy2015	GPS	incubation	9	144.96	7	80	8932	100	3906	19530	73.48
Gentoo Peng.	South Shetland	Admiralty Bay	PTT	brood-guard	23	14.99	7	55	390	100	947	4736	99.98
Gentoo Peng.	South Shetland	Admiralty Bay	PTT	crèche	37	20.04	7	60	486	100	947	4736	99.91
Gentoo Peng.	South Shetland	King George Island	GPS	brood-guard	42	14.91	7	55	518	90.48	476	2152	98.95
Gentoo Peng.	South Orkney	Signy Island (N Point)	GPS	incubation	6	35.92	7	75	865	100	263	1315	97.97

[1] – Based on the minimum percentage of birds using each site, set as 20% (see methods), multiplied by the colony size (Table 1);

[2] – Based on the maximum percentage of birds using each site, multiplied by the colony size (Table 1)

[3] – Small colony (less than 10,000 pairs and less than 1% of the global population), so site does not qualify as IBA

[4] – Asymptote in bootstrap analysis not reached (representativeness test; Lascelles et al., 2016)

Table S2.2: IBA criteria applied in the marine areas (adapted from BirdLife International, 2010 and Donald et al., in press)

	**Criterion**	**Description**
**A1: Globally Threatened Species**	*the site is known or thought to regularly hold significant numbers of a globally threatened species*	The site qualifies if it is known, estimated or thought to hold a population of a species categorized on the IUCN Red List as globally threatened (Critically Endangered, Endangered or Vulnerable). The regular presence at a site of a Critically Endangered or Endangered species, irrespective of population size, may be sufficient to propose it as an IBA. Population-size thresholds for site selection for Vulnerable species are agreed regionally on a species-by-species basis (typically equating to 1% of the global population). The list of globally threatened species is maintained and updated annually for IUCN by BirdLife International (http://datazone.birdlife.org/species/search)
**A4: Congregations**	*The site is known or thought to hold congregations of ≥1% of the global population of one or more species on a regular or predictable basis.*	Sites can qualify whether thresholds are exceeded simultaneously or cumulatively, within a limited period. In this way, the criterion covers situations where a rapid turnover of birds takes place (including, for example, for migratory species).<br><br>Sub-criteria (to be applied at a regional level):<br>***A4i****: the site is known or thought to hold, on a regular basis, ≥ 1% of a biogeographic population of a congregatory waterbird species.*<br>***A4ii****: the site is known or thought to hold, on a regular basis, ≥ 1% of the global population of a congregatory seabird or terrestrial species.*<br>***A4iii****: the site is known or thought to hold, on a regular basis, ≥ 20,000 waterbirds or ≥ 10,000 pairs of seabirds of one or more species.*<br>***A4iv****: the site is known or thought to exceed thresholds set for migratory species at bottleneck sites.*

References

Augé, A.A., Dias, M.P., Lascelles, B., Baylis, A.M.M., Black, A., Boersma, P.D., Catry, P., Crofts, S., Galimberti, F., Granadeiro, J.P., Hedd, A., Ludynia, K., Masello, J.F., Montevecchi, W., Phillips, R.A., Pütz, K., Quillfeldt, P., Rebstock, G.A., Sanvito, S., Staniland, I.J., Stanworth, A., Thompson, D., Tierney, M., Trathan, P.N. and Croxall, J.P. (2018). Framework for mapping key areas for marine megafauna to inform Marine Spatial Planning: The Falkland Islands case study. Marine Policy 92: 61–72. https://doi.org/10.1016/j.marpol.2018.02.017

BirdLife International (2009). Draft guidelines for using seabird tracking data to inform the identification of marine IBAs. Results from a workshop held in CNRS, Chize, France, July 2009. BirdLife international report. Cambridge, UK

BirdLife International (2010). Marine Important Bird Areas toolkit: standardised techniques for identifying priority sites for the conservation of seabirds at sea. BirdLife International, Cambridge UK. Version 1.2: February 2011

Börger, L., Franconi, N., De Michele, G., Gantz, A., Meschi, F., Manica, A., Lovari, S. and Coulson, T. (2006). Effects of sampling regime on the mean and variance of home range size estimates. Journal of Animal Ecology 75: 1393–1405. doi:10.1111/j.1365-2656.2006.01164.x

Fauchald, P. and Tveraa, T. (2003) Using first-passage time in the analysis of area-restricted search and habitat selection. Ecology 84: 282-288.

Freitas, C. (2012). argosfilter: Argos locations filter. R package version 0.63. https://CRAN.R-project.org/package=argosfilter

Johnson, D.S. (2017). crawl: Fit Continuous-Time Correlated Random Walk Models to Animal Movement Data. R package version 2.1.1. https://CRAN.R-project.org/package=crawl

Lascelles, B.G, Taylor, P., Miller, M., Dias, M.P., Oppel, S. et al. (2016) Applying global criteria to tracking data to define important areas for marine conservation. Diversity & Distributions 22: 422-431.

Soanes, L.M., Arnould, J.P.Y., Dodd, S.G., Sumner, M.D. and Green, J.A. (2013). How many seabirds do we need to track to define home-range area? Journal of Applied Ecology 50: 671–679

Suryan, R.M., Sato, F., Balogh, G.R., Hyrenbach, K.D., Sievert, P.R. and Ozaki, K. (2006) Foraging destinations and marine habitat use of short-tailed albatrosses: a multi-scale approach using

first passage time analysis. Deep Sea Research Part II: Topical Studies in Oceanography 53: 370–386.

Warwick-Evans, V., Ratcliffe, N., Lowther, A., Manco, F., Ireland, L., Clewlow, H. and Trathan, P.N. (2018). Using preferred habitat models for chinstrap penguins (*Pygoscelis Antarctica)* to advise krill fisheries management during the penguin breeding season. Diversity and Distribution. DOI: 10.1111/ddi.12817

Weimerskirch, H., Pinaud, D., Pawlowski, F. and Bost, C.-A. (2007) Does prey capture induce area-restricted search? A fine-scale study using GPS in a marine predator, the wandering albatross. The American Naturalist 170: 734–743

Wood, A.G, Naef-Daenzer, B., Prince, P.A. and Croxall, J.P. (2000). Quantifying habitat use in satellite-tracked pelagic seabirds: application of kernel estimation to albatross locations. Journal of Avian Biology 31: 278–286
