# Supplementary material for: Identification of marine Important Bird and Biodiversity Areas for penguins around the South Shetland Islands and South Orkney Islands
Source: Ecol Evol. 2018 Oct 12;8(21):10520–9. doi: 10.1002/ece3.4519 (PMC6238121; doi:10.1002/ece3.4519)

Supporting Information 3: MAPS WITH THE BOUNDARIES OF CANDIDATE MARINE IBAS

Identification of marine Important Bird and Biodiversity Areas for penguins around the South Shetland Islands and South Orkney Islands, by MP Dias, APB Carneiro, V Warwick-Evans, C Harris, K Lorenz, B Lascelles, H Clewlow, MJ Dunn, JT Hinke, J-H Kim, N Kokubun, F Manco, N Ratcliffe, M Santos,A Takahashi,W Trivelpiece,P Trathan.

<u>Candidate marine IBA boundaries for **Chinstrap penguins** – datasets used to test parameters.</u> Background maps show the intensity of area use (by different birds); red polygons represent the candidate marine IBA boundaries. Colonies, breeding stages and values used in the analyses are shown in the titles (smooth=*h-value*, udl=UD%, th=PT).

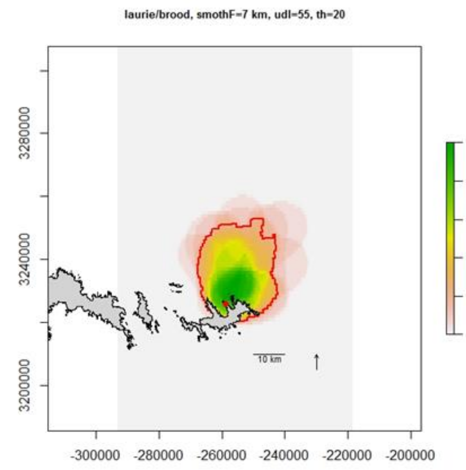

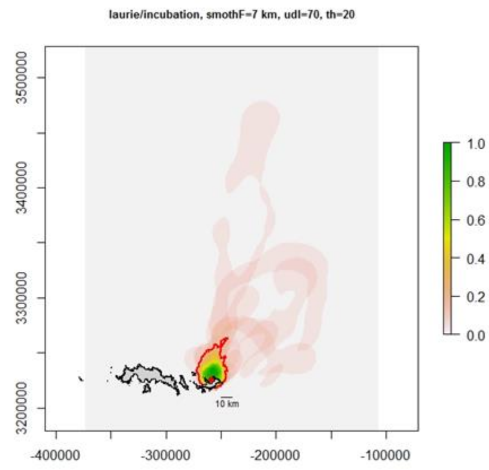

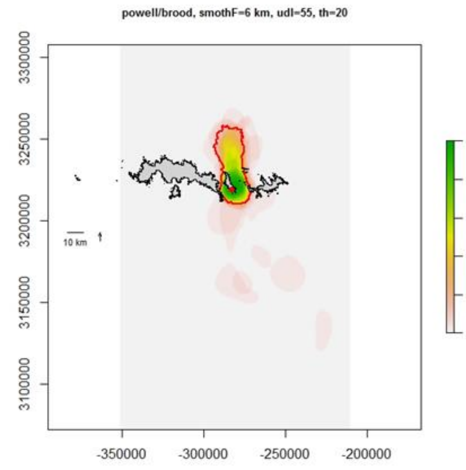

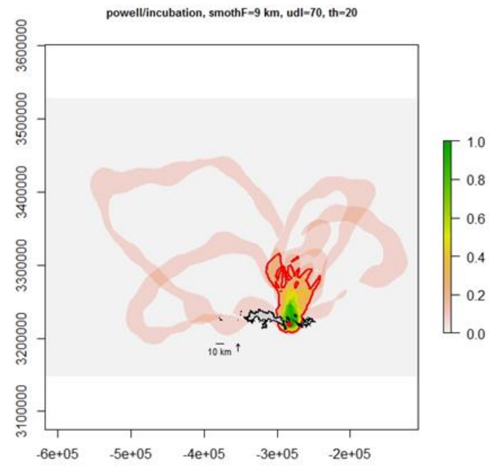

Candidate marine IBA boundaries for **Chinstrap penguins** – datasets used to test parameters (cont.).

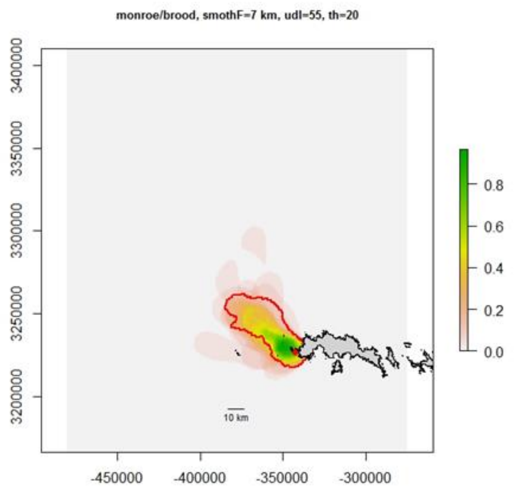

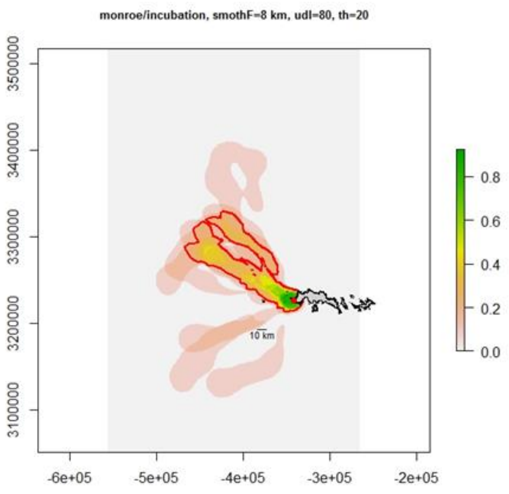

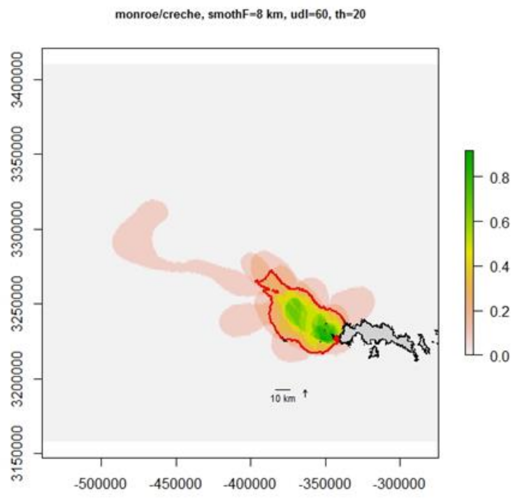

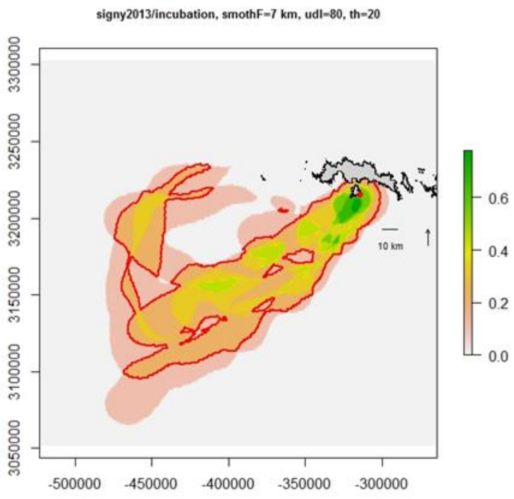

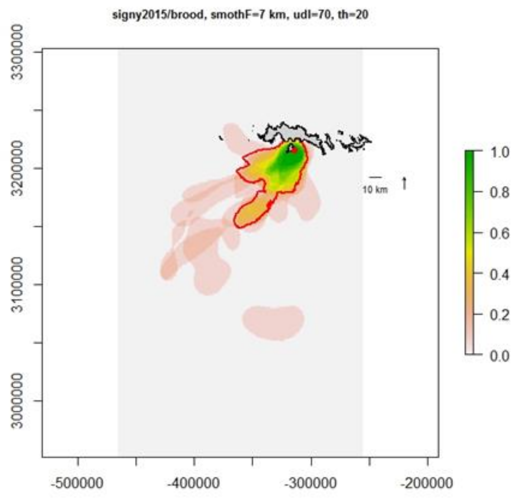

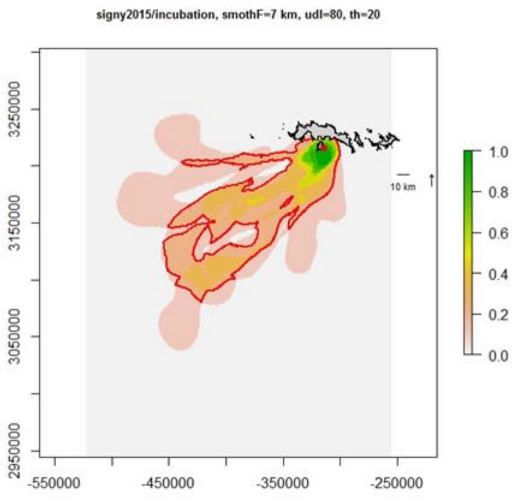

Candidate marine IBA boundaries for **Chinstrap penguins** – additional datasets. Background maps show the intensity of area use (by different birds); red polygons represent the candidate marine IBA boundaries. Colonies, breeding stages and values used in the analyses are shown in the titles (smooth=*h-value*, udl=UD%, th=PT).

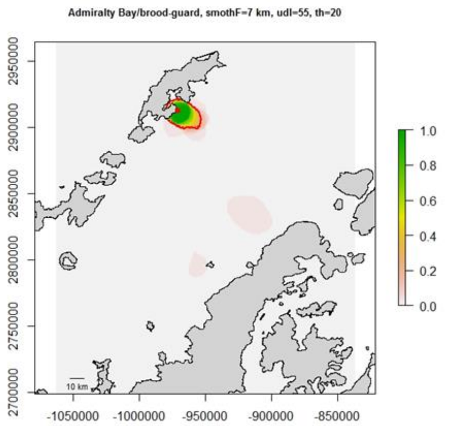

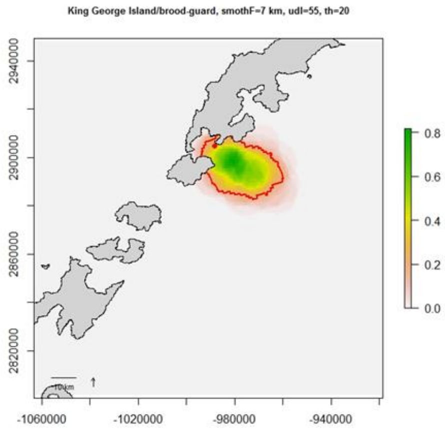

Candidate marine IBA boundaries for **Gentoo penguins.** Background maps show the intensity of area use (by different birds); red polygons represent the candidate marine IBA boundaries. Colonies, breeding stages and values used in the analyses are shown in the titles (smooth=*h-value*, udl=UD%, th=PT).

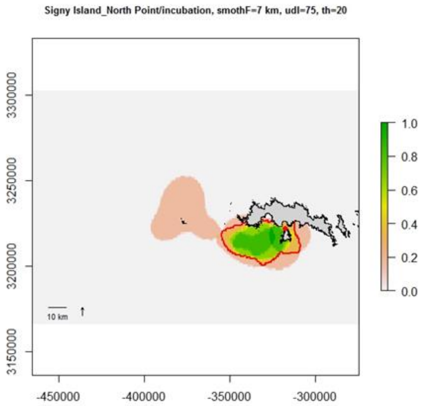

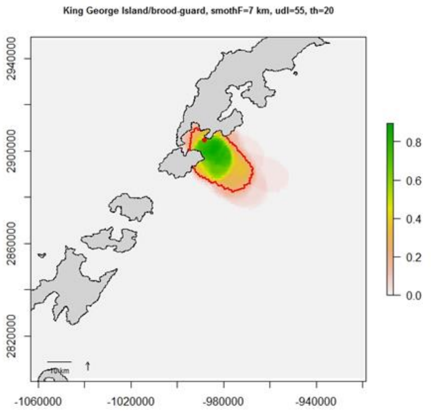

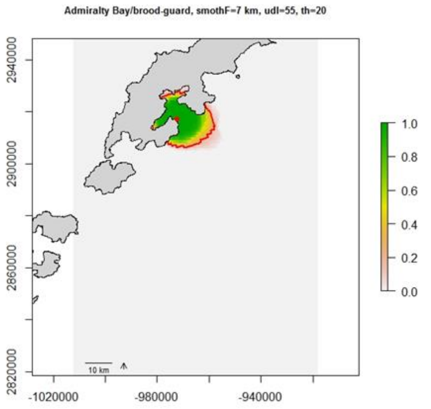

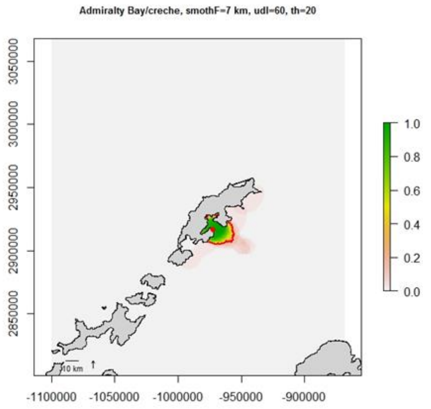

Candidate marine IBA boundaries for **Adélie penguins.** Background maps show the intensity of area use (by different birds); red polygons represent the candidate marine IBA boundaries. Colonies, breeding stages and values used in the analyses are shown in the titles (smooth=*h-value*, udl=UD%, th=PT).

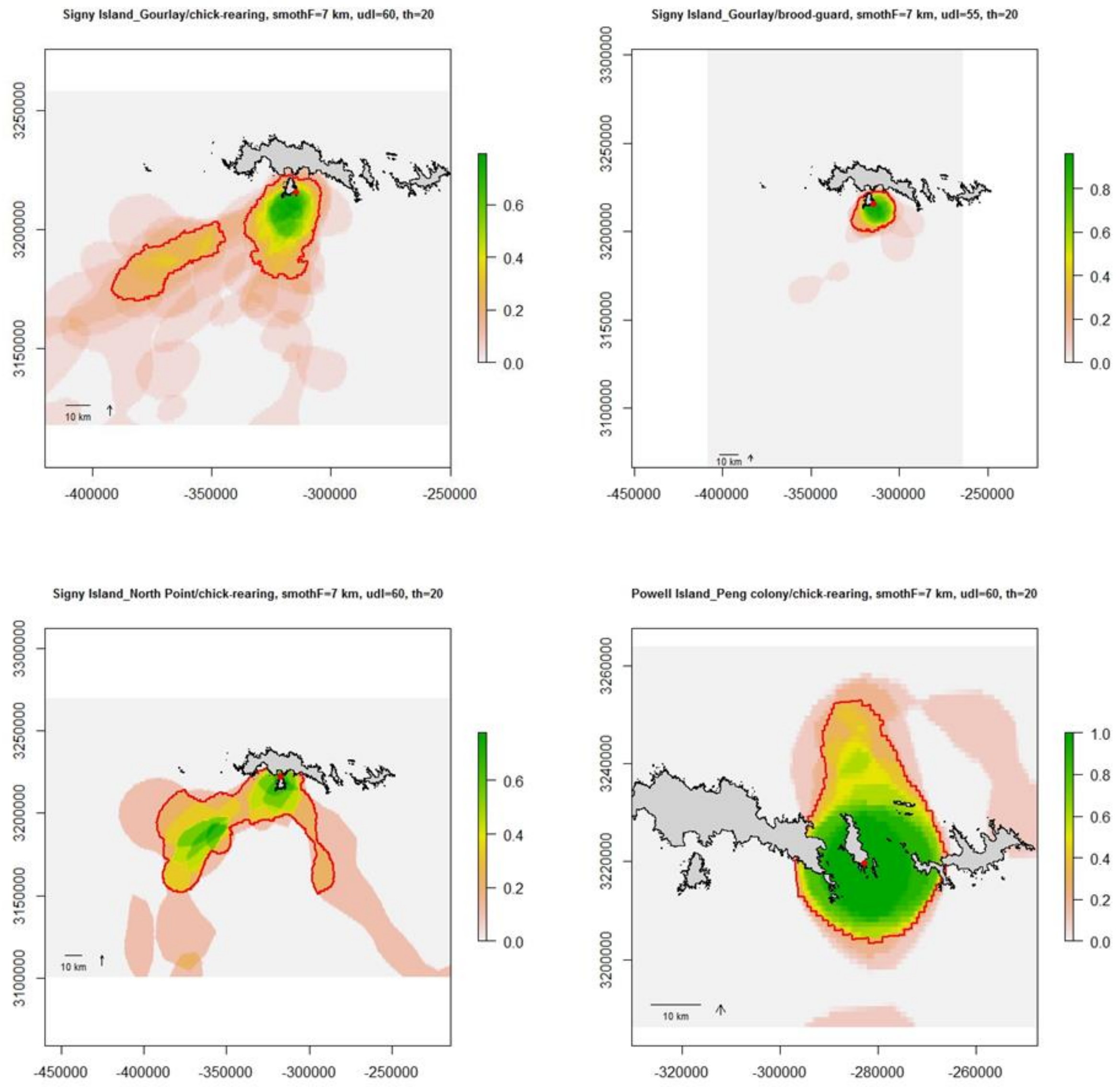

Candidate marine IBA boundaries for **Adélie penguins** (cont.).

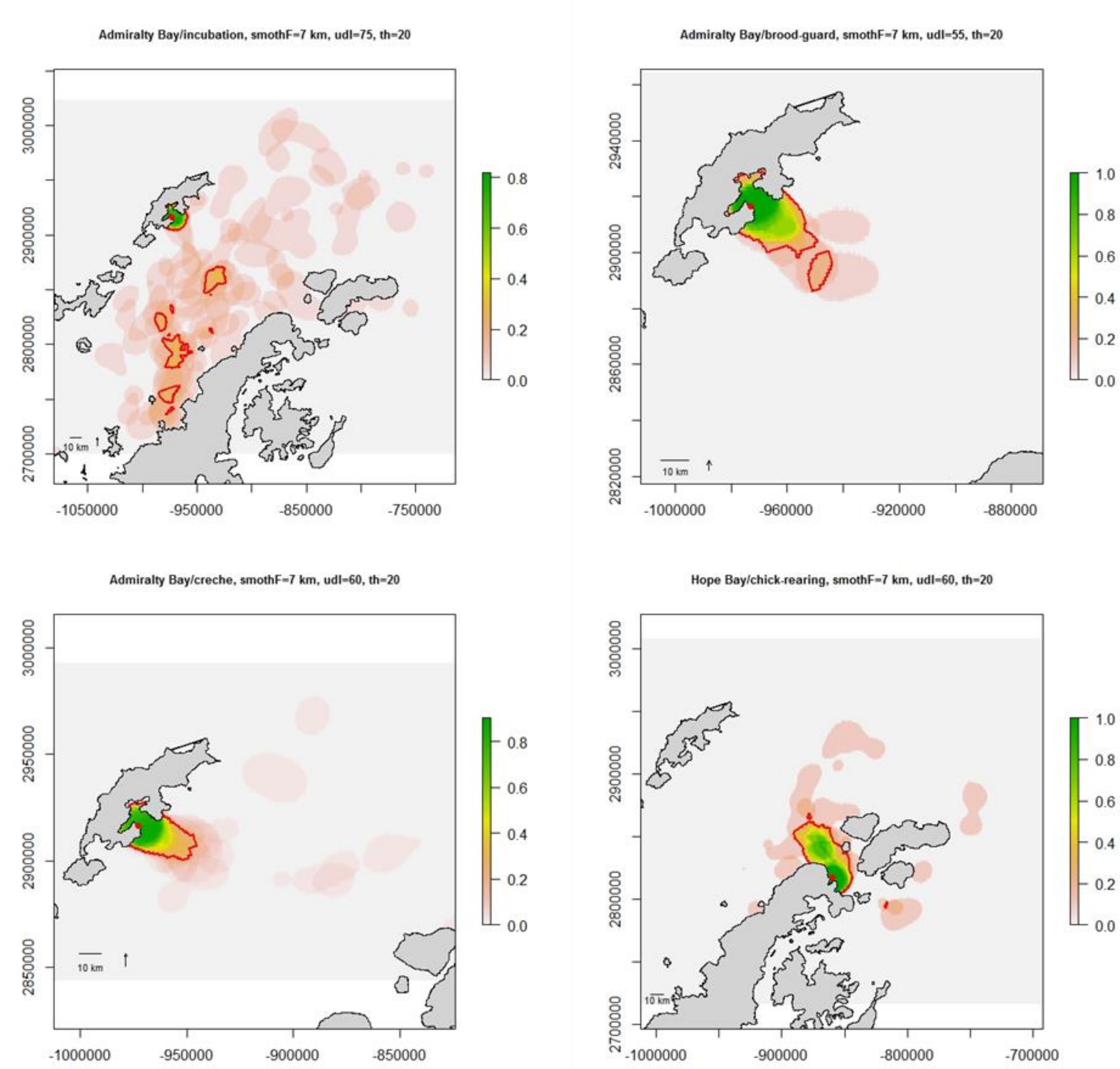

Supplement: Supplementary file 3 [file ECE3-8-10520-s003.pdf]
