# Supplementary material for: Identification of marine Important Bird and Biodiversity Areas for penguins around the South Shetland Islands and South Orkney Islands
Source: Ecol Evol. 2018 Oct 12;8(21):10520–9. doi: 10.1002/ece3.4519 (PMC6238121; doi:10.1002/ece3.4519)
Supplement: Supplementary file 4 [file ECE3-8-10520-s004.pdf]

Supporting Information 4: FRAMEWORK FOR A PENGUIN-SPECIFIC MARINE IBA PROTOCOL

Identification of marine Important Bird and Biodiversity Areas for penguins around the South Shetland Islands and South Orkney Islands, by MP Dias, APB Carneiro, V Warwick-Evans, C Harris, K Lorenz, B Lascelles, H Clewlow, MJ Dunn, JT Hinke, J-H Kim, N Kokubun, F Manco, N Ratcliffe, M Santos,A Takahashi,W Trivelpiece,P Trathan.

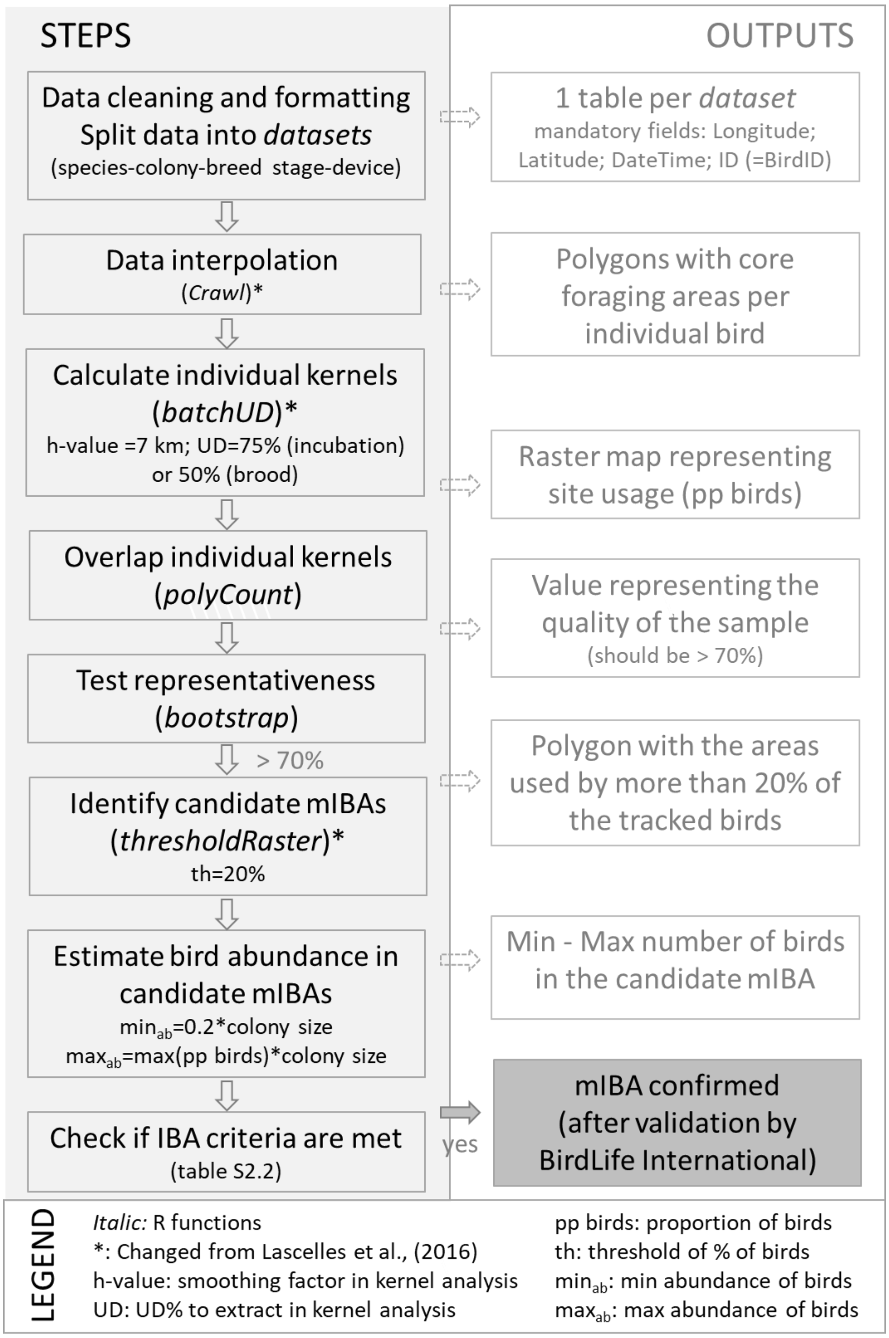

Fig S4.1. Schema with the steps and corresponding outputs of the penguin-specific marine IBA protocol (see also Figure S4.2)

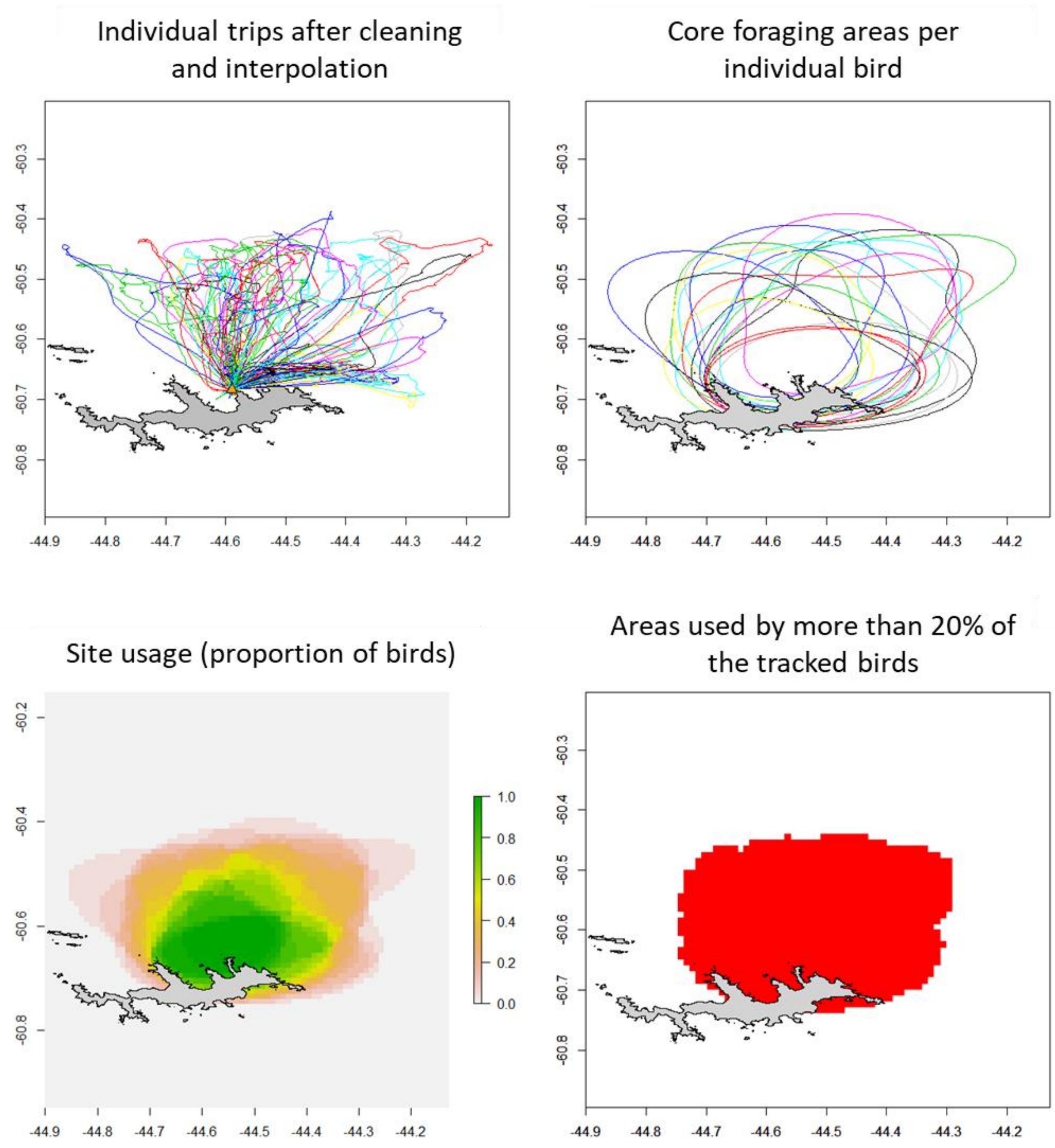

Fig S4.2. Examples of some main outputs resulting from the application of the mIBA protocol for penguins (See also Figure S4.1). Dataset: Chinstrap penguin-Laurie-brood guard-GPS

Table S4.1 Brief explanation of some key technical terms used throughout the text, related with the collection and analysis of tracking data

**Term**	**Description**
Dataset	Unique combination of tracking data collected for a single species in a specific colony, during a unique breeding stage and using a certain type of device.
Breeding stage	Period of the annual cycle when the data were collected (e.g. incubation, brood-guard, crèche, chick-rearing, non-breeding, sabbatical). In this study we've analysed data collected during incubation, brood-guard and crèche, and only for breeding adults.
Device	Type of logger deployed on the birds to record their geographic position and respective time stamp (e.g., GPS, ARGOS/PTT and Geolocators). In this study we've analysed data collected with GPS and PTT devices.
GPS	Global Positioning System
PTT- Argos	Platform Transmitter Terminal
h-value	Smoothing factor for the kernel analysis
UD%	Kernel Utilisation Distribution
PT	Threshold percentage of the population used to identify the boundaries if IBA candidates

R Code (adaptation from Lascelles et al., 2016; updated version of Lascelles et al., 2016 code can be found at https://github.com/steffenoppel/seabirds/blob/master/mIBA_functions_par.r)

```
###############
#crawl interpolates the locations using a correlated random walk approach

# require(devtools)
# install_version("crawl", "1.5")

library(crawl)   #to fit Kalman filter models
library(trip)    #to prepare GPS data
library(maptools)
library(sp)
library(rgdal)

#x1 is a data frame of raw location points for an individual bird (an additional loop is needed to run multiple individuals at the same time)
#it must contain the columns; Date (which is time and date), Lon and Lat (in decimal degrees) and bird ID
#format times
x1$Time<-as.POSIXct(strptime(paste(x1$Date), "%Y-%m-%d %H:%M:%S"), tz = "GMT")
#change times to hours since first GPS fix
x1$Time2<-as.numeric(difftime(x1$Time,min(x1$Time),units="hours"))
#save the start time for later so we can convert time back
 timeon<-as.POSIXct(x1$Time[1])
## remove completely-duplicated rows
x1 <- x1[!duplicated(x1), ]
## order the rows by time
x1 <- x1[order(x1$Time), ]

#set the error for the gps as zero, and the land as zero. If using PTT data, set the PTT location class
#errors here
x1$ErrX<-0
x1$ErrY<-0
x1$Land<-0
## fudge duplicated times
x1$Time<- adjust.duplicateTimes(x1$Time, x1$bird)

 ##########################################################################
  ##Apply McConnell speed filter in trip package to remove duff fixes #
  ##########################################################################
  x2<-data.frame(lat=x1$Lat,lon=x1$Lon,Date=x1$Time,id=x1$bird)
  #Create coordinate variable
  coordinates(x2) <- c("lon","lat")
  #create trip object
  tr <- trip(x2,c("Date","id"))
options(warn=1)
  #McConnell Speed filter; ignore coordinates warning as data are lon lat
  x1$Filter <- speedfilter(tr, max.speed = 8)
```

```
#remove filtered coordinates
  x1<-subset(x1,x1$Filter==TRUE)


  #create dataframe with only the required data
  xy<-data.frame(longitude=x1$Lon,latitude=x1$Lat,time=x1$Time2,id=x1$bird,dry=x1$Land,
              errX=x1$ErrX,errY=x1$ErrY)

# State starting values
initial.values <- list(a1.x=c(xy$longitude[1], 0, 0), a1.y=c(xy$latitude[1], 0, 0),
                   P1.x=diag(c(1, 1, 1)),
                   P1.y=diag(c(1, 1, 1)))
 #fix the parameters

fixP=c(NA, 1, NA, 1, NA, NA, NA,NA)
  #thet=c(-4,3,2,3)

  # Check out the parameters
  #displayPar(mov.model=~1, err.model=list(x=~errX, y=~errY), drift.model=T, data=xy,fixPar=fixP)

  #removes last fit: if this one doesn't converge predictions will not be made from previous model
  if(exists("fit")){rm(fit)}

  #fit model: ensure you get a "converged" confirmation at the end of the run
  #and no "Cannot calculate covariance matrix" warning.
  #if such problems arise try increasing the maxit values and trying several times

  #break the loop if I get warning messages
  #You can turn warnings into errors with:
    options(warn=2)


  fit <- crwMLE(mov.model=~1, err.model=list(x=~errX, y=~errY), drift.model=T,
             data=xy, coord=c("longitude", "latitude"), polar.coord=TRUE,
             Time.name="time", initial.state=initial.values,
             fixPar=fixP,
             control=list(maxit=1000,trace=1, REPORT=10),
             initialSANN=list(maxit=6000, trace=1, REPORT=1))

  #then keep warnings as warnings

    options(warn=1)
  #create time intervals: 5 mins here
predTime <- seq(ceiling(min(xy$time)), floor(max(xy$time)), 1/12)
#simulate locations
  predObj <- crwPredict(object.crwFit=fit, predTime, speedEst=TRUE, flat=TRUE)
  timenum<-as.numeric(timeon)
#convert the time back to a normal format
  predObj$Time3<-timenum+(predObj$time*3600)
  predObj$time4<-as.POSIXct(predObj$Time3, origin="1970-01-01")
```

```
  #plot map of best fit track (red line) and locations at times of original fixes (blur dots)
  crwPredictPlot(predObj, 'map')
  #plot(SO, col="gray", add=T)  #add map (SO is a shapefile of the south Orkney Islands)
  #subset so we just get interesting stuff
  predObj<-predObj[,c(1:9,15,21,32)]

  write.csv(predObj,"……..”),row.names=F)
  #remove observed locations and create dataframes with columns of interest
  bf_track<-subset(predObj,predObj$locType=="p")
  bf_track<-
data.frame(Time=predTime,Lon=bf_track$mu.x,Lat=bf_track$mu.y,bird=bird,time4=bf_track$time4)
 write.csv(bf_track,"….”,row.names=F)

#########################
batchUD
##########################################################################################
#

## Phil Taylor & Mark Miller, 2011

## batchUD calculates the Utilisation Distribution for groups of data in
## a larger dataset. The function relies on Adehabitat package for the
## calculations but returns the UD as SpatialPolygons so they can be exported
## as shapefiles and be more versatile in the script.

## DataGroup must be either a DataFrame or SpatialPointsDataFrame with Latitude,
## Longitude and ID as fields. UD will be calculated for each unique ID value and
## each row should be a location. For each UD to be comparable, the data should be
## regularly sampled or interpolated.
## Scale should be the smoothing factor to be used in the Kernel Density Estimation
## and should be provided in Km.
## UDLev should be the quantile to be used for the Utilisation Distribution.

## UPDATED BY STEFFEN OPPEL ON 16 Dec 2016 to fix orphaned holes in output geometry
## UPDATED BY STEFFEN OPPEL ON 23 Dec 2016 to switch to adehabitatHR
## UPDATED BY ANA CARNEIRO AND STEFFEN OPPEL 31 JAN 2017 to same4all=T
## UPDATED BY MARIA DIAS 28 JUL 2018 to fix scale at 7 km


batchUD <- function(DataGroup, Scale = 7, UDLev = 50)          # change UDLev accordingly to breed stage
   {
   require(sp)
   require(maptools)
   require(rgdal)
   require(adehabitatHR)   ### adehabitat is deprecated, switched to adehabitatHR on 23 Dec 2016
   require(geosphere)

   if(!"Latitude" %in% names(DataGroup)) stop("Latitude field does not exist")
   if(!"Longitude" %in% names(DataGroup)) stop("Longitude field does not exist")
   if(!"ID" %in% names(DataGroup)) stop("ID field does not exist")

   if(class(DataGroup)!= "SpatialPointsDataFrame")     ## convert to SpatialPointsDataFrame and project
   {
   mid_point<-data.frame(centroid(cbind(DataGroup$Longitude, DataGroup$Latitude)))
```

```
   DataGroup.Wgs <- SpatialPoints(data.frame(DataGroup$Longitude, DataGroup$Latitude),
proj4string=CRS("+proj=longlat + datum=wgs84"))
   DgProj <- CRS(paste("+proj=laea +lon_0=", mid_point$lon, " +lat_0=", mid_point$lat, sep=""))
   DataGroup.Projected <- spTransform(DataGroup.Wgs, CRS=DgProj)
   DataGroup <- SpatialPointsDataFrame(DataGroup.Projected, data = DataGroup)
   }else{DgProj<-DataGroup@proj4string}

   DataGroup$X <- DataGroup@coords[,1]
   DataGroup$Y <- DataGroup@coords[,2]


    ###### ENSURE THAT ONLY TRACKS WITH 5 OR MORE LOCATIONS ARE RETAINED (added 27 Feb 2017) ####
    UIDs <- names(which(table(DataGroup$ID)>5))      ### previous implementation restored
    #DataGroup@data$count<-1
    #nlocs<-aggregate(count~ID,DataGroup@data,sum)
    #retain<-as.character(nlocs$ID[nlocs$count>5])     ### kernelUD will fail for any ID with <5 locations
    DataGroup<-DataGroup[(DataGroup@data$ID %in% UIDs),]
    DataGroup@data$ID<-droplevels(as.factor(DataGroup@data$ID))     ### encountered weird error when
unused levels were retained (27 Feb 2017)

   UIDs <- unique(DataGroup$ID)
   #note<-0     removed loop to check whether >5 data points exist per trip - considered unnecessary
   #KDE.Sp <- NULL
   TripCoords<-SpatialPointsDataFrame(DataGroup,
data=data.frame(ID=DataGroup@data$ID,TrackTime=DataGroup@data$TrackTime))
   TripCoords@data$TrackTime<-NULL
   Ext <- (min(coordinates(TripCoords)[,1]) + 3 * diff(range(coordinates(TripCoords)[,1])))
   if(Ext < (Scale * 1000 * 2)) {BExt <- ceiling((Scale * 1000 * 3)/(diff(range(coordinates(TripCoords)[,1]))))} else
{BExt <- 5} #changed from 3 to 5 on 23 Dec 2016 to avoid 'too small extent' error

   ### NEED TO EXPLORE: CREATE CUSTOM GRID TO feed into kernelUD (instead of same4all=T)

KDE.Surface <- adehabitatHR::kernelUD(TripCoords, h=(Scale * 1000), grid=1000, extent=BExt, same4all=TRUE)
                 ## newer version needs SpatialPoints object and id no longer required in adehabitatHR, also
removed 'extent' as it caused problems
KDE.Sp <- adehabitatHR::getverticeshr(KDE.Surface, percent = UDLev,unin = "m", unout = "km2") ##    syntax
differs from older version; THIS FUNCTION CAN FAIL WHEN same4all=FALSE


   KDE.Sp@proj4string <- DgProj
   KDE.Wgs <- spTransform(KDE.Sp, CRS=CRS("+proj=longlat +ellps=WGS84"))
   Tbl <- data.frame(Name_0 = rep(1, length(UIDs)), Name_1 = 1:length(UIDs), ID = UIDs)
   row.names(Tbl) <- UIDs
   KDE.Spdf <- SpatialPolygonsDataFrame(KDE.Sp, data=Tbl)

   plot(KDE.Spdf, border=factor(UIDs))

    ##### OVERLAYS IN THE polyCount FUNCTION WILL NOT WORK IF THE POLYGONS CONTAIN HOLES OR ARE
ORPHANED
    ## simple fix to remove holes from polygon object
    va90a <- spChFIDs(KDE.Spdf, paste(KDE.Spdf$Name_0, KDE.Spdf$Name_1, KDE.Spdf$ID, sep = ""))
    va90a <- va90a[, -(1:4)]
    va90_pl <- slot(va90a, "polygons")
    va90_pla <- lapply(va90_pl, checkPolygonsHoles)
    p4sva <- CRS(proj4string(va90a))
    vaSP <- SpatialPolygons(va90_pla, proj4string = p4sva)
```

```
     va90b <- SpatialPolygonsDataFrame(vaSP, data = as(va90a, "data.frame"))   ### this returns an empty data
frame
     va90b@data<-KDE.Spdf@data                                                 ### this adds the original data back into the data
frame - may not work if entire polygons are removed


   return(va90b)     ## changed from KDE.Spdf to replace with cleaned version
   }


#####bootstrap
###########################################################################################
############

## Phil Taylor & Mark Miller, 2012

## this script Iteratively subsamples a dataset of tracking data, investigating the
## affect of sample size. At each iteration the data is split, one half is used as the
## 'training' data and the 50%UD is calculated from this. The second half is used as
## 'testing' and the proportion of it, captured within the 50%UD is calculated.
## A perfect dataset would tend towards 0.5.
## By fitting a trend line to this relationship we can establish the sample size at which
## any new data would simply add to the existing knowledge. This script indicates how close to
## this value the inputted data are.

## DataGroup must be a dataframe or SpatialPointsDataFrame with Latitude, Longitude and ID as fields.
## Scale determines the smoothing factor used in the kernel analysis.
## Iteration determines the number of times each sample size is iterated.

## REVISED BY Steffen Oppel in 2015 to facilitate parallel processing
## updated to adehabitatHR by Steffen Oppel on 27 Dec 2016
## changed to same4all=TRUE on 4 Feb 2017

## REVISED in 2017 to avoid error in nls function of singular gradient
## added mean output for inclusion value even if nls fails

## UPDATED BY MARIA DIAS 28 JUL 2018 to allow different UD values and output the representativeness value


bootstrap <- function(DataGroup, Scale=100, Iteration=50, UDLev=50)              #change UDLev
  {

  require(sp)
  require(geosphere)
  require(rgdal)
  require(adehabitatHR)
  require(foreach)
  require(doParallel)
  require(parallel)

  if(!"Latitude" %in% names(DataGroup)) stop("Latitude field does not exist")
  if(!"Longitude" %in% names(DataGroup)) stop("Longitude field does not exist")
  if(!"ID" %in% names(DataGroup)) stop("ID field does not exist")

  if(class(DataGroup)!= "SpatialPointsDataFrame")     ## convert to SpatialPointsDataFrame and project
   {
   mid_point<-data.frame(centroid(cbind(DataGroup$Longitude, DataGroup$Latitude)))
```

```
  DataGroup.Wgs <- SpatialPoints(data.frame(DataGroup$Longitude, DataGroup$Latitude), proj4string=CRS("+proj=longlat + datum=wgs84"))
  DgProj <- CRS(paste("+proj=laea +lon_0=", mid_point$lon, " +lat_0=", mid_point$lat, sep=""))
  DataGroup.Projected <- spTransform(DataGroup.Wgs, CRS=DgProj)
  DataGroup <- SpatialPointsDataFrame(DataGroup.Projected, data = DataGroup)
  }else{DgProj<-DataGroup@proj4string}

  DataGroup$X <- DataGroup@coords[,1]
 DataGroup$Y <- DataGroup@coords[,2]
 BoundBox <- bbox(DataGroup)
 UIDs <- unique(DataGroup$ID)
 Ntrips <- length(UIDs)
 Nloop<- seq(1,(Ntrips-1),ifelse(Ntrips>100,10,1))
 DoubleLoop <- data.frame(SampleSize = rep(Nloop,each=Iteration), Iteration=rep(seq(1:Iteration),length(Nloop)))
 LoopNr <- seq(1:dim(DoubleLoop)[1])
 #UDLev <- 50                                   # updated by MARIA DIAS

#setup parallel backend to use 4 processors
cl<-makeCluster(detectCores())
registerDoParallel(cl)
Result<-data.frame()

Result <- foreach(LoopN=LoopNr, .combine = rbind, .packages=c("sp","adehabitatHR","geosphere","rgdal")) %dopar% {

  N<-DoubleLoop$SampleSize[LoopN]
  i<-DoubleLoop$Iteration[LoopN]
  Coverage <- NULL
  Inclusion <- NULL
  History <- NULL

  Output <- data.frame(SampleSize = N, InclusionMean = 0,Iteration=i)

   RanNum <- sample(UIDs, N, replace=F)
   SelectedCoords <- coordinates(DataGroup[DataGroup$ID %in% RanNum,])
   NotSelected <- DataGroup[!DataGroup$ID %in% RanNum,]
   Temp <- data.frame(SelectedCoords[,1], SelectedCoords[,2])
   Ext <- (min(Temp[,1]) + 3 * diff(range(Temp[,1])))
   if(Ext < (Scale * 1000 * 2)) {BExt <- ceiling((Scale * 1000 * 3)/(diff(range(Temp[,1]))))} else {BExt <- 5}
   Temp <- SpatialPoints(Temp,proj4string=DgProj)      ### added because adehabitatHR requires SpatialPoints object

   KDE.Surface <- adehabitatHR::kernelUD(Temp, h=(Scale * 1000), grid=1000, extent=BExt, same4all=TRUE)
                    ## newer version needs SpatialPoints object and id no longer required in adehabitatHR
  try(     ## inserted to avoid function failing if one iteration in bootstrap has crazy extent
   KDE.UD <- adehabitatHR::getverticeshr(KDE.Surface, percent = UDLev,unin = "m", unout = "km2"), silent = TRUE)                    ## syntax differs from older version
   #KDE.Spl <- kver2spol(KDE.UD)     ## deprecated, newer function would be khr2estUDm, but not required
   #KDE.Spl@proj4string <- DgProj    ## no longer necessary after update to adehabitatHR
   if("KDE.UD" %in% ls()){Overlain <- over(NotSelected, KDE.UD)   ## changed from KDE.Spl
   Output$InclusionMean <- length(which(!is.na(Overlain$area)))/nrow(NotSelected)    ## updated because overlay will not yield predictable number
   }             ## inserted if statement to avoid function failing when one KDE.UD cannot be calculated
  return(Output)
  }
```

```
## stop the cluster
stopCluster(cl)

  par(mfrow=c(1,1), mai=c(1,1,1,1))
  #Result <- Output[1:nrow(Output)-1,]
  write.table(Result,"bootout_temp.csv", row.names=F, sep=",")
  try(M1 <- nls((Result$InclusionMean ~ (a*Result$SampleSize)/(1+b*Result$SampleSize)), data=Result, start=list(a=1,b=0.1)), silent = TRUE)
 if ('M1' %in% ls()){       ### run this only if nls was successful
  PredData <- data.frame(SampleSize = unique(Result$SampleSize))
  Result$pred<-predict(M1)
  P2 <- aggregate(pred~SampleSize, Result, FUN=mean)
  P2$sd <- aggregate(InclusionMean~SampleSize, Result, FUN=sd)[,2]
  plot(InclusionMean~SampleSize, data=Result, pch=16, cex=0.2, col="darkgray", ylim=c(0,1), xlim=c(0,max(PredData)), ylab="Inclusion", xlab="SampleSize")
  yTemp <- c((P2[,2] + 0.5*P2[,3]), rev(P2[,2] - 0.5*P2[,3]))
  xTemp <- c(P2$SampleSize, rev(P2$SampleSize))
  polygon(x=xTemp, y=yTemp, col="gray93", border=F)
  points(InclusionMean~SampleSize, data=Result, pch=16, cex=0.2, col="darkgray")
  lines(P2, lty=1,lwd=2)
  Asymptote <- (summary(M1)$coefficients[1]/summary(M1)$coefficients[2])
  RepresentativeValue <- max(P2$pred)/Asymptote*100
  print(RepresentativeValue)
  Result$RepresentativeValue <- RepresentativeValue
  text(x=0, y=1,paste(round(RepresentativeValue,2), "%", sep=""), cex=2, col="gray45", adj=0)
}else{RepresentativeValue <- mean(Result$InclusionMean[Result$SampleSize==max(Result$SampleSize)])   ### if nls is unsuccessful then use mean output for largest sample size
  Result$RepresentativeValue <- (RepresentativeValue/(UDLev/100))*100}      ## added by Jono Handley to convert to same scale as nls output

  print(ifelse(exists("M1"),"nls (non linear regression) successful, asymptote estimated for bootstrap sample.",
      "WARNING: nls (non linear regression) unsuccessful, likely due to 'singular gradient', which means there is no asymptote. Data may not be representative, output derived from mean inclusion value at highest sample size. Check bootstrap output csv file"))

  return(RepresentativeValue) # updated by MARIA DIAS

  }



##polyCount
############################################################################################################

## Phil Taylor & Mark Miller, 2012

## polyCount calculates the number of overlapping Polygons. The calculation is
## done by overlapping the polygons within a grid and counting the number
## falling within each gridcell. The Res parameter sets the size of the grid
## (in decimal degrees) to be used. Smaller grids will allow for more detailed
## counts but will slow computing time. The function returns a raster object with
## values showing the proportion of Polys (i.e. nPolys/total nPolys) overlapping
## each cell. The raster extent is set at the bounding limits of the Polys or, when
## data crosses the dateline, set to the northern and southern most limits of the
## Polys but longitudinally crossing the circumference of the world.
```

```
## Polys must be a SpatialPolygonsDataFrame of the polygons to be counted.
## Res must be a numeric object indicating the resolution in decimal degrees.

## Steffen Oppel revision on 14 Dec 2016 - removed + (Res * 100) from NCol in L. 664
## Steffen Oppel revision on 16 Dec 2016: fixed the point overlay problem by using a proper grid instead
## Steffen Oppel revision on 27 Dec 206: ensured that output was projected in WGS84

## version 1.2    05-04-2012

polyCount <- function(Polys, Res = 0.1)
  {

  require(raster)
  require(maps)

  if(!class(Polys) %in% c("SpatialPolygonsDataFrame", "SpatialPolygons")) stop("Polys must be a
SpatialPolygonsDataFrame")
  if(is.na(projection(Polys))) stop("Polys must be projected")

  Poly.Spdf <- spTransform(Polys, CRS=CRS("+proj=longlat +ellps=WGS84"))
  DgProj <- Polys@proj4string

  DateLine <- Poly.Spdf@bbox[1,1] < -178 & Poly.Spdf@bbox[1,2] > 178
  if(DateLine == TRUE) {print("Data crosses DateLine")}

  UDbbox <- bbox(Poly.Spdf)
  if(DateLine == TRUE)  {UDbbox[1,] <- c(-180,180)}
  BL <- floor(UDbbox[,1])                # + (Res/2) - removed on 16 Dec 2016 because it results in some polygons
outside the grid
  TR <- ceiling(UDbbox[,2])
  NRow <- ceiling(sqrt((BL[1] - TR[1])^2)/Res)
  NCol <- ceiling(sqrt((BL[2] - TR[2])^2)/Res) #+ (Res * 100)                  ### THIS LINE CAUSES
PROBLEMS BECAUSE IT GENERATES LATITUDES >90 which will cause spTransform to fail
  Grid <- GridTopology(BL, c(Res,Res), c(NRow, NCol))
    newgrid<-SpatialGrid(Grid, proj4string = CRS("+proj=longlat + datum=wgs84"))
    spol <- as(newgrid, "SpatialPolygons")                                      ###
this seems to create an orphaned hole
    SpGridProj <- spTransform(spol, CRS=DgProj)
    GridIntersects <- over(SpGridProj, Polys)
    SpGridProj<-    SpatialPolygonsDataFrame(SpGridProj,    data    =    data.frame(ID=GridIntersects$ID,
row.names=sapply(SpGridProj@polygons,function(x) x@ID)))
    SpGridProj <- subset(SpGridProj, !is.na(SpGridProj@data$ID))
  #SpGrid <- SpatialPoints(Grid, proj4string = CRS("+proj=longlat + datum=wgs84"))
  #SpdfGrid    <-    SpatialPointsDataFrame(SpGrid,    data.frame(Longitude=SpGrid@coords[,1],
Latitude=SpGrid@coords[,2]))
  #SpGridProj <- spTransform(SpdfGrid, CRS=DgProj)
  #GridIntersects <- over(SpGridProj, Polys)
  #SpGridProj@data$Intersects$ID <- GridIntersects$ID
  #SpGridProj    <-    subset(SpGridProj,    !is.na(SpGridProj@data$Intersects$ID))    ###
SpGridProj[!is.na(SpGridProj@data$Intersects$ID),]        ###
  plot(SpGridProj)

  Count <- 0
  for(i in 1:length(Polys))
    {
    TempB <- Polys[i,]
```

```
    Temp <- over(SpGridProj, TempB)[,1]     ### inserted based on Matthew Carroll's advice; MAY NEED TO SWAP
arguments in 'over'?
    Temp[is.na(Temp)] <- 0
    Temp[Temp > 0] <- 1
    Count <- Count + Temp
    #Prop <- Count/i                      ### removed to improve efficiency
    }
  Prop <- Count/length(Polys)     ### removed from loop over polys as it only needs to be calculated once
  #GridIntersects$inside<-as.numeric(as.character(GridIntersects$ID))    ### this only works for numeric trip_id!!
  #GridIntersects$Prop <- 0
  #GridIntersects$Prop[!is.na(GridIntersects$inside)] <- Prop    #[,1] removed based on Matthew Carroll's advice,
because fixed in L. 678
  SpGridProj@data$Prop <- Prop
  SpGridOUT <- spTransform(SpGridProj, CRS=CRS("+proj=longlat +ellps=WGS84"))   ### show output in WGS84
  SGExtent <- extent(SpGridOUT)
  RT <- raster(SGExtent, ncols=as.double(NCol), nrows=as.double(NRow))
  WgsRas <- (rasterize(x=SpGridOUT,y=RT, field = "Prop"))

  plot(WgsRas, asp=1)
  maps::map("world", add=T, fill=T, col="darkolivegreen3")      ## to avoid conflict with purrr
  projection(WgsRas) <- CRS("+proj=longlat + datum=wgs84")
  return(WgsRas)
  }


##thresholdRaster
##################################################################################################
############

## Phil Taylor & Mark Miller, 2012

## thresholdRaster applies a threshold to a raster, and isolates any areas above
## that threshold value. Converting the raster values to polygons is difficult and
## so this part takes some time. The function returns a SpatialPolygonsDataFrame
## containing the polygons that are above threshold, and with an attributes table
## holding each sites Maximum raster value.

## CountRas must be a raster object with values 0 - 1.
## Threshold must be a number indicating the percentage value to be used
## as the threshold.
## UPDATED BY MARIA DIAS 28 JUL 2018: set threshold at 20%

thresholdRaster <- function(CountRas, Threshold = 20)
    {

    require(raster)
    require(maps)
    require(geosphere)

    plot(CountRas, asp=1)
    maps::map("world", add=T, fill=T, col="darkolivegreen3")
    Threshold <- Threshold/100
    RasSites <- CountRas >= Threshold
    plot(RasSites, asp=1, col=rev(heat.colors(25)))
    maps::map("world", add=T, fill=T, col="darkolivegreen3")      ### to avoid conflict with purrr

    if(length(which(getValues(CountRas) > Threshold)) < 1)
```

```
   {
   Mid   <-   c(bbox(CountRas)[1,1]+(as.numeric(bbox(CountRas)[1,2])-   as.numeric(bbox(CountRas)[1,1]))/2,
midPoint(bbox(CountRas)[,2], bbox(CountRas)[,1])[2])
   text(Mid, "No Site Identified", cex=1.25)
   stop("No cells were above the threshold value")
   }

  #Cells <- rasterToPolygons(CountRas, fun=function(x) {x>Threshold})
  Cells <- rasterToPolygons(clump(CountRas>Threshold), dissolve=TRUE)   ## suggested by Ian Cleasby, as more
efficient
  DateLine <- Cells@bbox[1,1] < -178 & Cells@bbox[1,2] > 178
  if(DateLine == TRUE)    {Cells <- spTransform(Cells, CRS=DgProj)}

  Sites <- Cells                                   ## removed based on suggestion from Ian Cleasby: dissolve(Cells)
  ifelse(DateLine == TRUE, projection(Sites) <- DgProj, projection(Sites) <- "+proj=longlat + datum=wgs84")
  Sites <- spTransform(Sites, CRS=CRS("+proj=longlat + datum=wgs84"))
  SiteTable  <-  data.frame(SiteID  =  names(Sites),  MaxPerc  =  round(raster::extract(CountRas,  Sites,
fun=max)*100,2))    ## to avoid conflict with tidyverse
  Sites <- SpatialPolygonsDataFrame(Sites, data=SiteTable)
  print(SiteTable)
  return(Sites)
  }

##### THIS FUNCTION MAY NOT BE NECESSARY AS 'clump' and 'dissolve=T' in the 'RasterToPolygon' function
can perform this calculation
dissolve <- function(Cells)
{
require(sp)
require(rgeos)
require(maptools)
CellsAv <- Cells
plot(Cells)
j <- 0
while(length(CellsAv) > 0)
 {
 j <- j + 1

 Cell1 <- CellsAv[1,]
 CellsAv <- CellsAv[-1,]
 if(length(CellsAv) < 1)
  {
  CellsMerge <- spChFIDs(Cell1, as.character(j))
  if(j == 1) {Sites <- CellsMerge} else
  Sites <- spRbind(Sites, CellsMerge)
  next
  }
 CellsNr <- which(gTouches(Cell1, CellsAv, byid=T))
 if(length(CellsNr) < 1)
  {
  CellsMerge <- spChFIDs(Cell1, as.character(j))
  if(j == 1) {Sites <- CellsMerge} else
  Sites <- spRbind(Sites, CellsMerge)
  next
  }
 CellsSel <- CellsAv[as.double(CellsNr),]
 CellsMerge <- gUnion(Cell1, CellsSel)
 CellsAv <- CellsAv[-as.double(CellsNr),]
```

```
  if(length(CellsAv) < 1)
   {
   CellsMerge <- spChFIDs(CellsMerge, as.character(j))
   if(j == 1) {Sites <- CellsMerge} else
   Sites <- spRbind(Sites, CellsMerge)
   next
   }
  CellsNr <- which(gTouches(CellsMerge, CellsAv, byid=T))
  while(length(CellsNr) > 0)
   {
   CellsSel <- CellsAv[as.double(CellsNr),]
   CellsMerge <- gUnion(CellsMerge, CellsSel)
   plot(CellsMerge, add=T, col=2)
   CellsAv <- CellsAv[-as.double(CellsNr),]
   if(length(CellsAv) < 1) break
   CellsNr <- which(gTouches(CellsMerge, CellsAv, byid=T))
   }
  CellsMerge <- spChFIDs(CellsMerge, as.character(j))
  if(j == 1) {Sites <- CellsMerge} else
  Sites <- spRbind(Sites, CellsMerge)
  plot(Sites, add=T, col=2)
  }
plot(Sites, col=names(Sites), add=T)
return(Sites)
}
```
